# Supplementary material for: Minimum dietary diversity and its associated factors among children aged 6–23 months in Senegal: Evidence from the demographic and health survey, 2023
Source: PLoS One. 2025 Sep 11;20(9):e0332215. doi: 10.1371/journal.pone.0332215 (PMC12425297; doi:10.1371/journal.pone.0332215)
Supplement: Attachment — Submitted filename: shakya-referee_report(plos_one).pdf [file pone.0332215.s002.pdf]

Referee Report

The manuscript, “Minimum Dietary Diversity and Its Associated Factors Among Children Aged 6-23 Months in Senegal: Evidence from Demographic and Health Survey, 2023” identifies key variables significantly associated with adequate minimum dietary diversity among children in Senegal. The author utilizes the most recent 2023 DHS survey data for Senegal and employs a multilevel mixed-effects logistic regression model to identify these key variables. The paper finds that media exposure (at both the individual and community levels), household wealth index, breastfeeding status, child’s age, and the number of under-5 children are significantly associated with minimum dietary diversity.

Below are my questions and comments on the manuscript:

Major Questions / Comments:

1. It is unclear what the contribution of the paper is to the literature on dietary diversity for children in Senegal.
   a) Factors that determine minimum dietary diversity for children in Senegal, as well as Sub-Saharan Africa, are well documented (see for e.g., Thorne-Lyman et al. 2020; Aboagye et al. 2021). Is the contribution of this paper that it utilizes the most recent DHS data? If so, the manuscript would benefit from discussions on how dietary diversity in Senegal has changed, and what new information can be identified using this most recent survey.
   b) Or, is the motivation that the paper identifies both individual and community level media exposure as a key determinant of children having adequate minimum dietary diversity? Linking media exposure to dietary diversity is relatively new for Senegal but has been explored for Ethiopia (Jalata and Asefa 2022) and other Sub-Saharan countries (Belay et al. 2022). Discussions on why media exposure is a key factor for dietary diversity need to be made clear in both the Introduction and Results sections.
2. The key attribute of this paper is identifying the variables significantly linked to children with adequate minimum dietary diversity. While the paper does use a comprehensive set of individual and community level characteristics, these are not exhaustive. For instance, mother’s occupation, father’s characteristics, local agriculture, distance to major cities, and local climate are some key variables that could also impact minimum dietary diversity. Models don’t have to include all possible variables, but a better explanation of the choice of independent variables needs to be provided. At the moment, the paper lacks justification for why certain variables were chosen, and more importantly, why others were not.
   a) On a similar note, defining poverty levels for community by taking 50% of women who belonged to the lowest and worst wealth quintiles is also not justified. This seems like an arbitrary number that might not really reflect poverty levels in Senegal. Justification for this would also help readers understand subsequent results.

3. Media exposure is defined as members having access to newspapers, watching television, or listening to the radio (line 147). These variables seem to be strongly correlated with household wealth and access to basic amenities. The fact that media exposure is strongly associated with adequate minimum dietary diversity is not surprising, as its likely that households with media exposure are simply wealthier households. At the moment, both the wealth index and media exposure are significant, but I am concerned that these two variables merely reflect wealthier households. A stronger justification is needed to explain why these two variables are distinct. What is specific about 'media exposure' that explains why children achieve minimum dietary diversity that is not related to household wealth index?
4. Regarding the empirical methods, the paper assesses model strength using log-likelihood (line 179). This value is further used to justify the selection of models (line 225). However, to the best of my knowledge, log-likelihood values improve with more variables. Therefore, the fact that Model III (Table 2) has the lowest log-likelihood is not surprising, as Model III includes the highest number of independent variables. As in Comment #2, better justification for the choice of model is needed. Why is Model III considered rather than any alternative model with more additional variables?
5. Recent studies have highlighted that in Senegal, seasonality plays a key role in determining a child's dietary diversity (see for e.g., Shakya, Bevis and Thorne-Lyman 2024; Thorne-Lyman et al. 2021). During the harvest season, households have higher incomes and food availability, leading to better child diets. Similarly, Ramadan is a key festival in Senegal, which directly affects household diets. Information on when the DHS surveys were conducted (e.g., how many households were surveyed in Jan, Feb, …, Dec), would help readers understand how seasonality might bias minimum dietary diversity estimates. For example, if most households were surveyed during the harvest season – when food and income levels are high – then dietary diversity among children in Senegal is likely overestimated. Discussing this in the Limitation section would be helpful.

Minor Questions / Comments

There are several typos and statements that need more justification. A few examples are highlighted below:

- Line 17: I think the software is "STATA". The manuscript is missing an A.
- Line 42: "caregiver should engage with their children to acknowledge their hunger cues", is a bit unclear. I am not sure what this means.
- Line 49: This sentence talks about the links between stunting and diet. There are many other key papers on this topic and should cite a few more seminal papers on this topic.
- Line 73: Similarly, this sentence talks about the importance of animal-sourced food. A lot of work on this has already been done and would benefit from citing these too.

- Line 77: MDD was 30.8%. Which year was this data for? The paper's MDD is around 23%. There seems to be a big difference between these two MDD.
- Line 93: What are EDS surveys? I could not find explanation for this.
- Line 106: To the best of my knowledge, KR stands for Kids Recode.
- Line 254: MDD scores for 32 Sub-Saharan country is 25.1%, but for 3 Sub-Saharan country it is 23.2%. Not sure what this means.
- Line 272-274: This claim needs supporting evidence (papers or reports).

References

Aboagye, R.G., A.-A. Seidu, B.O. Ahinkorah, F. Arthur-Holmes, A. Cadri, L.K. Dadzie, J.E. Hagan, O. Eyawo, and S. Yaya. 2021. "Dietary Diversity and Undernutrition in Children Aged 6–23 Months in Sub-Saharan Africa." *Nutrients* 13(10):3431.

Belay, D.G., F.M. Aragaw, R.E. Teklu, S.M. Fetene, W.D. Negash, D.B. Asmamaw, E.A. Fentie, T.G. Alemu, H.B. Eshetu, and E.S. Shewarega. 2022. "Determinants of Inadequate Minimum Dietary Diversity Intake Among Children Aged 6–23 Months in Sub-Saharan Africa: Pooled Prevalence and Multilevel Analysis of Demographic and Health Survey in 33 Sub-Saharan African Countries." *Frontiers in Nutrition* 9. Available at: https://www.frontiersin.org/journals/nutrition/articles/10.3389/fnut.2022.894552/full [Accessed March 6, 2025].

Jalata, D.D., and B.G. Asefa. 2022. "Trends and Determinants of Dietary Diversity in Children Aged 6–59 Months in Ethiopia: Analysis of 2005–2016 Demographic and Health Survey." *Current Developments in Nutrition* 6(10):nzac135.

Shakya, K.S., L.E.M. Bevis, and A.L. Thorne-Lyman. 2024. "Diet and disease: Examining the seasonal determinants of children's health in Senegal." *Food Policy* 129:102735.

Thorne-Lyman, A., H. Kuo, A. Kc, S. Manohar, B. Shrestha, K. West Jr., R. Klemm, and R. Heidkamp. 2020. "How Seasonal Is the Minimum Dietary Diversity for Children Indicator? An Investigation in Three Countries on Three Continents: Senegal, Nepal and Peru." *Current Developments in Nutrition* 4:nzaa046_070.

Thorne-Lyman, A.L., L.E.M. Bevis, H. Kuo, S. Manohar, B. Shrestha, A. KC, R.D. Klemm, and R.A. Heidkamp. 2021. "Season of Data Collection of Child Dietary Diversity Indicators may Affect Conclusions about Longer Term Trends in Peru, Senegal and Nepal." *Current Developments in Nutrition* (nzab095). Available at: https://doi.org/10.1093/cdn/nzab095 [Accessed July 20, 2021].
