# Supplementary material for: Minimum dietary diversity and its associated factors among children aged 6–23 months in Senegal: Evidence from the demographic and health survey, 2023
Source: PLoS One. 2025 Sep 11;20(9):e0332215. doi: 10.1371/journal.pone.0332215 (PMC12425297; doi:10.1371/journal.pone.0332215)
Supplement: Attachment — Submitted filename: PONE-D-24-35929_R1-rev-Omas-3May25.pdf [file pone.0332215.s004.pdf]

PLOS ONE

Minimum Dietary Diversity and its Associated Factors among Children Aged 6–23 Months in Senegal: Evidence from the Demographic and Health Survey, 2023

--Manuscript Draft--

Manuscript Number:	PONE-D-24-35929R1
Article Type:	Research Article
Full Title:	Minimum Dietary Diversity and its Associated Factors among Children Aged 6–23 Months in Senegal: Evidence from the Demographic and Health Survey, 2023
Short Title:	Minimum Dietary Diversity and its Associated Factors among Children Aged 6–23 Months in Senegal
Corresponding Author:	Enyew Getaneh Mekonen<br>University of Gondar College of Medicine and Health Sciences<br>Gondar, ETHIOPIA
Keywords:	Minimum dietary diversity<br>DHS<br>Multilevel analysis<br>Young children<br>Senegal
Abstract:	Background: Children between the ages of 6 and 23 months should be provided a variety of foods to ensure that their nutrient needs are satisfied, according to World Health Organization guidelines for feeding children. In Senegal, lower dietary diversity and an inability to meet nutrient requirements raise the risk of anemia and other disorders caused by a lack of funds and resources, which are essential for consuming a nutrient-rich diet. The objective of this study was to assess minimum dietary diversity and its associated factors among children aged 6–23 months in Senegal using the most recent demographic and health survey data (2023).<br>Methods: A cross-sectional study was conducted in Senegal using data from the most recent continuous demographic and health survey, 2023. A total of 2,995 children aged 6–23 months were included in the study. Using STATA Version 14 software, the data were extracted, cleaned, and analyzed. A multilevel mixed-effects logistic regression analysis was used to determine the factors associated with meeting minimum dietary diversity. Finally, variables with a p-value less than 0.05 were declared statistically significant.<br>Results: In the present study, the proportion of children aged 6-23 months who meet minimum dietary diversity in Senegal was 23.61% (95% CI: 22.12, 25.16). Factors like media exposure [AOR = 1.51; 95% CI (1.08, 2.13)], household wealth index [AOR = 1.84; 95% CI (1.24, 2.74)], ANC visits [AOR = 1.50; 95% CI (1.05, 2.13)], current breastfeeding status [AOR = 1.82; 95% CI (1.37, 2.43)], age of the child [AOR = 7.47; 95% CI (5.43, 10.3)], number of under-5 children in the household [AOR = 3.97; 95% CI (1.38, 11.4)], and community-level media exposure [AOR = 1.41; 95% CI (1.03, 1.93)] were significantly associated with meeting minimum dietary diversity.<br>Conclusions: Nearly one in four children aged 6 to 23 months achieved minimum dietary diversity in Senegal. Children from mothers with media exposure, wealthier households, attending 4+ antenatal care visits, currently breastfeeding, older children, and no under-5 children in the household were associated with higher odds of meeting minimum dietary diversity. Therefore, improving media access, women's empowerment, enhancing antenatal care service utilization, encouraging continued breastfeeding, and giving prior attention to younger children aged 6 to 11 months and mothers who had under-five children in the household are strongly recommended.
Order of Authors:	Enyew Getaneh Mekonen
Response to Reviewers:	I have addressed all the comments and attached them as a separate file.
Additional Information:	
Question	Response
Financial Disclosure	The author(s) received no specific funding for this work.

Powered by Editorial Manager® and ProduXion Manager® from Aries Systems Corporation

Enter a financial disclosure statement that describes the sources of funding for the work included in this submission. Review the submission guidelines for detailed requirements. View published research articles from *PLOS ONE* for specific examples.<br><br>This statement is required for submission and **will appear in the published article** if the submission is accepted. Please make sure it is accurate.<br><br>**Funded studies**<br>Enter a statement with the following details:<br>• Initials of the authors who received each award<br>• Grant numbers awarded to each author<br>• The full name of each funder<br>• URL of each funder website<br>• Did the sponsors or funders play any role in the study design, data collection and analysis, decision to publish, or preparation of the manuscript?<br><br>Did you receive funding for this work?	
**Competing Interests**<br><br>Use the instructions below to enter a competing interest statement for this submission. On behalf of all authors, disclose any competing interests that could be perceived to bias this work—acknowledging all financial support and any other relevant financial or non-financial competing interests.<br><br>This statement is required for submission and **will appear in the published article** if the submission is accepted. Please make sure it is accurate and that any funding sources listed in your Funding Information later in the submission form are also declared in your Financial Disclosure statement.	The authors have declared that no competing interests exist.

View published research articles from *PLOS ONE* for specific examples.<br><br>**NO authors have competing interests**<br><br>Enter: *The authors have declared that no competing interests exist.*<br><br>**Authors with competing interests**<br><br>Enter competing interest details beginning with this statement:<br><br>*I have read the journal's policy and the authors of this manuscript have the following competing interests: [insert competing interests here]*<br><br>* typeset	
**Ethics Statement**<br><br>Enter an ethics statement for this submission. This statement is required if the study involved:<br><br>• Human participants<br>• Human specimens or tissue<br>• Vertebrate animals or cephalopods<br>• Vertebrate embryos or tissues<br>• Field research<br><br>Write "N/A" if the submission does not require an ethics statement.<br><br>General guidance is provided below. Consult the submission guidelines for detailed instructions. **Make sure that all information entered here is included in the Methods section of the manuscript.**	Permission was granted to download and use the data from http://www.dhs.program.com before conducting the study. Ethical clearance was obtained from the Institution Review Board of the DHS Program, ICF International. The procedures for DHS public-use data sets were approved by the Institution Review Board. Identifiers for respondents, households, or sample communities were not allowed in any way, and the names of individuals or household addresses were not included in the data files. The number for each EA in the data file does not have labels to show their names or locations. There were no patients or members of the public involved since this study used a publicly available data set.

**Format for specific study types**<br><br>**Human Subject Research (involving human participants and/or tissue)**<br>• Give the name of the institutional review board or ethics committee that approved the study<br>• Include the approval number and/or a statement indicating approval of this research<br>• Indicate the form of consent obtained (written/oral) or the reason that consent was not obtained (e.g. the data were analyzed anonymously)<br><br>**Animal Research (involving vertebrate animals, embryos or tissues)**<br>• Provide the name of the Institutional Animal Care and Use Committee (IACUC) or other relevant ethics board that reviewed the study protocol, and indicate whether they approved this research or granted a formal waiver of ethical approval<br>• Include an approval number if one was obtained<br>• If the study involved *non-human primates*, add *additional details* about animal welfare and steps taken to ameliorate suffering<br>• If anesthesia, euthanasia, or any kind of animal sacrifice is part of the study, include briefly which substances and/or methods were applied<br><br>**Field Research**<br><br>Include the following details if this study involves the collection of plant, animal, or other materials from a natural setting:<br>• Field permit number<br>• Name of the institution or relevant body that granted permission	
**Data Availability**<br><br>Authors are required to make all data underlying the findings described fully available, without restriction, and from the time of publication. PLOS allows rare exceptions to address legal and ethical concerns. See the PLOS Data Policy and FAQ for detailed information.	Yes - all data are fully available without restriction

A Data Availability Statement describing where the data can be found is required at submission. Your answers to this question constitute the Data Availability Statement and **will be published in the article**, if accepted.<br><br>**Important:** Stating 'data available on request from the author' is not sufficient. If your data are only available upon request, select 'No' for the first question and explain your exceptional situation in the text box.<br><br>Do the authors confirm that all data underlying the findings described in their manuscript are fully available without restriction?	
**Describe where the data may be found in full sentences. If you are copying our sample text, replace any instances of XXX with the appropriate details.**<br><br>• If the data are **held or will be held in a public repository**, include URLs, accession numbers or DOIs. If this information will only be available after acceptance, indicate this by ticking the box below. For example: *All XXX files are available from the XXX database (accession number(s) XXX, XXX.).*<br>• If the data are all contained **within the manuscript and/or Supporting Information files**, enter the following: *All relevant data are within the manuscript and its Supporting Information files.*<br>• If neither of these applies but you are able to provide **details of access elsewhere**, with or without limitations, please do so. For example:<br><br>*Data cannot be shared publicly because of [XXX]. Data are available from the XXX Institutional Data Access / Ethics Committee (contact via XXX) for researchers who meet the criteria for access to confidential data.*<br><br>*The data underlying the results presented in the study are available from (include the name of the third party*	The data from the DHS is publicly available online at https://dhsprogram.com/data/available-datasets.cfm.

Powered by Editorial Manager® and ProduXion Manager® from Aries Systems Corporation

and contact information or URL).<br>• This text is appropriate if the data are owned by a third party and authors do not have permission to share the data.<br><br>* typeset	
Additional data availability information:	

Powered by Editorial Manager® and ProduXion Manager® from Aries Systems Corporation

Manuscript Click here to access/download;Manuscript;Manuscript.docx

Minimum Dietary Diversity and its Associated Factors among Children Aged 6–23 Months in Senegal: Evidence from the Demographic and Health Survey, 2023

Enyew Getaneh Mekonen

Department of Surgical Nursing, School of Nursing, College of Medicine and Health Sciences, University of Gondar, Gondar, Ethiopia

enyewgetaneh12@gmail.com

Abstract

Background: Children between the ages of 6 and 23 months should be provided a variety of foods to ensure that their nutrient needs are satisfied, according to World Health Organization guidelines for feeding children. In Senegal, lower dietary diversity and an inability to meet nutrient requirements raise the risk of anemia and other disorders caused by a lack of funds and resources, which are essential for consuming a nutrient-rich diet. The objective of this study was to assess minimum dietary diversity and its associated factors among children aged 6–23 months in Senegal using the most recent demographic and health survey data (2023).

Methods: A cross-sectional study was conducted in Senegal using data from the most recent continuous demographic and health survey, 2023. A total of 2,995 children aged 6–23 months were included in the study. Using STATA Version 14 software, the data were extracted, cleaned, and analyzed. A multilevel mixed-effects logistic regression analysis was used to determine the factors associated with meeting minimum dietary diversity. Finally, variables with a p-value less than 0.05 were declared statistically significant.

Results: In the present study, the proportion of children aged 6-23 months who meet minimum dietary diversity in Senegal was 23.61% (95% CI: 22.12, 25.16). Factors like media exposure [AOR = 1.51; 95% CI (1.08, 2.13)], household wealth index [AOR = 1.84; 95% CI (1.24, 2.74)], ANC visits [AOR = 1.50; 95% CI (1.05, 2.13)], current breastfeeding status [AOR = 1.82; 95% CI (1.37, 2.43)], age of the child [AOR = 7.47; 95% CI (5.43, 10.3)], number of under-5 children in the household [AOR = 3.97; 95% CI (1.38, 11.4)], and community-level media exposure [AOR = 1.41; 95% CI (1.03, 1.93)] were significantly associated with meeting minimum dietary diversity.

Conclusions: Nearly one in four children aged 6 to 23 months achieved minimum dietary diversity in Senegal. Children from mothers with media exposure, wealthier households, attending 4+ antenatal care visits, currently breastfeeding, older children, and no under-5 children in the household were associated with higher odds of meeting minimum dietary diversity. Therefore, improving media access, women's empowerment, enhancing antenatal care service utilization, encouraging continued breastfeeding, and giving prior attention to younger children aged 6 to 11 months and mothers who had under-five children in the household are strongly recommended.

Keywords: minimum dietary diversity, DHS, multilevel analysis, young children, Senegal

Background

For children to grow and develop to the best extent possible, the proper meals must be consumed at the right times. When it comes to nutrition, the 1,000 days from conception to the child's second birthday are the most important (1). Children need to start eating their first foods around six months of age. Throughout the day, young children should be fed regularly, in sufficient amounts, and with a range of food groups in their meals that are high in nutrients. In addition to using clean

hands and dishes during meal preparation and serving, caregivers should engage with their children to acknowledge their nutrition requirements (2).

Children between the ages of 6 and 23 months should be provided a variety of foods to ensure that their nutrient needs are satisfied, according to World Health Organization (WHO) guidelines for feeding breastfed and non-breastfed children (3, 4). Variety in food groups is linked to better linear growth in early children (5). Micronutrient deficiencies may be more likely in a diet low in diversity, which could be detrimental to children's physical and mental development (6). According to a previous study, stunting was associated with little to no ingestion of nutrient-dense foods such as eggs, dairy products, fruits, and vegetables between the ages of 6 and 23 months (7).

Feeding infants and young children involves more than just making sure they get enough nourishment. Meals are social and cultural gatherings when young children learn about food preferences and dislikes, observe, and mimic behavior that will shape their eating habits for the rest of their lives (8). A child learns to handle food during meals and makes the connection between food tastes, appearances, and textures. Early child feeding should ideally encourage a child's independence, but it can also be used to control behavioral issues or give a child too much, which can have long-term negative effects on the child's nutrition and health (9). It's crucial to pay attention to how a child is fed in addition to what they eat (10).

To make it practical and relevant for Member State reporting, the WHO-UNICEF Technical Expert Advisory Group on Nutrition Monitoring (TEAM) suggested in June 2017 revising the minimum dietary diversity (MDD) indicator as defined by WHO (2008). The adjustment involved moving the MDD criterion from four of seven categories to five of eight groups and adding "breast milk" as an eighth dietary group (11). Therefore, if a child consumes five or more of the following food groups, their diet is considered diverse: breast milk; grains, roots, and tubers; legumes and

nuts; dairy products (milk, yogurt, cheese); flesh foods (meat, fish, poultry, liver, or other organs); eggs; vitamin A-rich fruits and vegetables; and other fruits and vegetables (12).

The relationship between dietary diversity and children's health is important, as it is a protective factor against low nutritional status, which can show up as underweight, which is a combination of stunting and wasting in children; wasting, which is an acute form of undernutrition; and stunting, which is chronic and long-term undernutrition (13-15). A diverse diet is linked to a decrease in undernutrition in children between the ages of six and twenty-three months (16). It was also observed that children who abstained from eating eggs, dairy products, and other fruits and vegetables had a higher probability of being underweight, severely stunted, and wasted (17).

In Senegal, lower dietary diversity and an inability to meet nutrient requirements raise the risk of anemia and other disorders caused by a lack of funds and resources, which are essential for consuming a nutrient-rich diet (18). The prevalence of MDD was 30.8% in Senegal, and the socioeconomic barriers to achieving dietary diversity were identified (19). The high prevalence of micronutrient malnutrition in Senegal makes it imperative to promptly analyze the nutritional adequacy of young children's meals in order to target the most vulnerable groups with suitable interventions (20). Studies conducted elsewhere showed that media exposure, household wealth index, age of the child, maternal education, number of families, number of under-five children, ANC visits, and residence were determinants of meeting MDD (19, 21-27). The objective of this study was to assess minimum dietary diversity and its associated factors among children aged 6–23 months in Senegal using the most recent demographic and health survey (DHS) data (2023).

Methods and materials

Data sources, study design, and setting

Cross-sectional data from the continuous DHS in Senegal in 2023 was used for the present study. The DHS-C 2023 is the fifth edition after the continuous surveys conducted in Senegal between 2012–2014, 2015–2016, 2017–2018, and 2019. Other DHS surveys were conducted in Senegal in 1986, 1992–93, 1997, 2005, and 2010. This edition of the survey used a nationally representative sample of 400 clusters and 8,800 households, with an expected number of 16,142 women aged 15-49 surveyed with success. All women aged 15 to 49 who are members of households or who have spent the night before the day of the survey in selected households are eligible for the survey. As with the previous DHS, the main purpose of the DHS-C 2023 is to gather information on the health of women and their young children, on fertility, on the knowledge and use of contraceptive methods, and on knowledge and attitudes towards sexually transmitted diseases. The results of the survey are representative for Senegal, for the urban environment and rural areas separately, and for each of the fourteen administrative regions. The National Agency for Statistics and Demography (NASD) has a computer file of Census Districts (DRs) created as a result of the General Population and Housing Census, Agriculture, and Livestock of 2013 (RGPHAE-2013). The datasets are publicly available from the DHS website, www.dhsprogram.com. The surveys are nationally representative of the country and population-based with large sample sizes (28).

Source and study populations

The youngest child, age 6–23 months, living with the mother in Senegal comprised the source population, whereas women in the selected primary sampling unit (PSU) comprised the study population. Weighted values, which were computed from the Kids Record (KR file) Senegal DHS

2023 datasets, were utilized to restore the representativeness of the sample data. Finally, this study comprised a weighted sample of 2,995 children aged 6-23 months.

Sampling procedure

The 2023 DHS-C sample is a random, stratified sample drawn at 2 degrees. The primary unit of survey is the DR as defined for the 2013 Census. Each region was separated into parts to form the sampling strata, and the sample was drawn independently from each sampling layer. A total of 28 sample strata were created. Inside each stratum, the DRs were sorted by administrative units below the region, i.e., departments and districts/communes, etc. This operation had introduced implicit stratification at the level of all administrative units below the region with an implicit stratification of the sample allocation proportional to unit size. Prior to the enumeration of households, each large DR having more than 200 households was divided into segments, of which only one was included in the sample. At the second level, in each of the DRs selected in the first, a fixed number of 22 households had been selected with a systematic probability draw from the newly established lists at the time of enumeration. Only the households drawn are interviewed. Replacements of pre-selected households were not allowed immediately, even if they were not for non-responding households, to avoid bias. Every DHS report could be accessed on the Measure DHS website, which included the comprehensive sampling procedure (29).

Variables of the study

Dependent variable

The outcome variable of this study was the minimum dietary diversity status of the child (adequate/inadequate). Children aged 6–23 months who consumed five or more foods and beverages out of the eight defined food groups during the day or night before the survey were

considered as meeting MDD (adequate), and inadequate otherwise. The eight food groups are a) breast milk, b) grains, white/pale starchy roots, tubers, and plantains, c) legumes and nuts, d) dairy products (infant formula, milk, yogurt, cheese), e) flesh foods (meat, fish, poultry, and liver/organ meats), f) eggs, g) vitamin A-rich fruits and vegetables, and h) other fruits and vegetables (30).

Independent variables

Independent variables from two sources (variables at the individual and community levels) were taken into consideration for this analysis, as DHS data are hierarchical in nature. Variables included at the individual-level were maternal age (15-24, 25-34, 35-49 years), maternal education (no education, primary, secondary & higher), marital status (unmarried, married), sex of the household head (male, female), total children ever born (1-3, 4-6, ≥7), media exposure (no, yes), wealth index (poor, middle, rich), ANC visits (none, 1-3 visits, 4+ visits), place of delivery (home, health facility), currently breastfeeding (no, yes), PNC checkup (no, yes), sex of the child (male, female), age of the child (6 – 11, 12 – 17, 18 – 23 months), birth order (first-born, 2-4, 5+), preceding birth interval (< 24 months, ≥ 24 months), and the number of under-5 children in the household (none, 1–2, 3+). Variables included at the community level were place of residence (urban, rural), community literacy (low, high), community-level poverty (low, high), and community media exposure (low, high) (Figure 1). These variables were included depending on their availability in the DHS and by reviewing previous literature.

Operational description of independent variables

Media exposure was established by combining three factors: reading newspapers, watching television, and listening to the radio. A woman was deemed to have been exposed to media if she had at least one kind of exposure.

Media exposure at the community level was measured using the percentage of women who had at least been exposed to one of the three media: television, radio, or newspapers. It was coded as "0" for low community-level media exposure (communities where less than 50% of women had at least one media exposure) and "1" for high community-level media exposure (communities where more than 50% of women had at least one media exposure).

The level of poverty in the community was calculated based on the percentage of women in the lowest and poorest quintiles, as obtained from the wealth index data. The poverty levels were classified as "0" for low (those where less than 50% of women belonged to the lowest wealth quintiles) and "1" for high (those where more than 50% of women belonged to the lowest wealth quintiles) communities.

Women's literacy at the community level was also measured using the percentage of women with at least a primary education. It was coded as "0" for low community-level women's education (communities where less than 50% of women had completed primary school) and "1" for high community-level women's education (communities where more than 50% of women completed primary education at the cluster level).

Data collection tools and quality assurance

Demographics and health surveys use interviewer-administered questionnaire methodologies to gather data through several questionnaire formats. The DHS guideline defined the missing values in the outcome variables in a straightforward manner (30). However, as complete case analysis is a preferable way to manage missing data in a cross-sectional study, variables with a missing value greater than 5% in explanatory variables were removed from further analysis. To guarantee quality, the data extractions were carried out by researchers with prior experience working with DHS data.

Model building for multi-level analysis

A multilevel logistic regression model was fitted because the DHS data is hierarchical and children were nested within a cluster, which goes against the independence and equal variance assumptions of ordinary logistic regression models. For the multi-level analysis, four models were fitted. The null model, which solely included the outcome variable, is employed to examine the variation in MDD throughout the cluster. Individual-level and community-level variables are included in the first (Model I) and second (Model II) multilevel models, respectively. The third model (Model III) fitted MDD simultaneously with factors at the individual and community levels. The log-likelihood and deviance test was used to compare the models with the standard logistic regression model, and the best-fitting model was determined by taking the highest log-likelihood and lowest deviance values of each model into account. Multicollinearity was checked using the variance inflation factor (VIF), and a variable is considered to be multicollinear if its VIF score is 10 or more (31). However, in this investigation, the mean VIF value of the final model was 1.60, and all variables had VIF values less than three. Finally, variables with a p-value of < 0.05 and 95% confidence intervals were declared statistically significant. Proportional Change in Variance (PCV), Intra-class Correlation Coefficient (ICC), and Median Odds Ratio (MOR) were used to evaluate the random effect utilized to measure the variation (32).

Ethical statement

Permission was granted to download and use the data from http://www.dhs.program.com before conducting the study. Ethical clearance was obtained from the Institutional Review Board of the DHS Program, ICF International. The procedures for DHS public-use data sets were approved by the Institutional Review Board. Identifiers for respondents, households, or sample communities were not allowed in any way, and the names of individuals or household addresses were not

included in the data files. The number for each EA in the data file does not have labels to show their names or locations. There were no patients or members of the public involved since this study used a publicly available data set.

Results

Individual- and community-level characteristics of study subjects

The mean age of mothers was 28.12 ± 0.13 years, and 42.27% of them fall in the age range of 25–34 years. More than half (54.79%) of mothers had no formal education, and only 5.51% of them were unmarried. More than three-fourths (79.33%) of mothers were from male-headed households, and 61.07% of them had three or fewer children ever born. More than half (57.83%) of mothers had poor household wealth status, and 82.57% of them had media exposure. More than two-thirds (67.91%) of mothers had four or more ANC visits during their pregnancy, and 81.97% of them currently breastfed their children. The majority (90.52%) of mothers gave birth at a health facility, and 57.77% of them had no postnatal checkup. The mean age of children was 14.18 ± 0.09 months, and 38.09% of them fall in the age range of 12–17 months. More than half (51.59%) of children were male, and 24.54% of them were first-born children. The preceding birth interval for almost all children (99.40%) was ≥ 24 months, and 55.73% of mothers had three or more under-five children in the household. More than one-third (35.43%) of mothers reside in urban areas, and 50.62% of them were from communities with low levels of literacy. Nearly two-thirds (66.58%) and 55.16% of mothers were from communities with high levels of media exposure and low levels of poverty, respectively [Table 1].

Prevalence of adequate minimum dietary diversity among children aged 6–23 months

In the present study, the proportion of children aged 6-23 months who meet minimum dietary diversity in Senegal was 23.61% (95% CI: 22.12, 25.16) [Figure 2]. The highest proportion of children who meet MDD were found in the Saint-Louis region (45.12%) and the lowest in the Sedhiou region (9.84%) [Figure 3]. The proportion of MDD was 89.68%, 31.40%, 73.27%, 79.92%, and 48.23% among children who had media exposure, rich households, attended 4+ ANC visits, were currently breastfeeding, and were aged 12-17 months, respectively [Table 2]. From the eight defined groups, breast milk was consumed by nearly 81% of young children, and only 6.61% of them consumed eggs [Figure 4].

Multi-level analysis of factors associated with MDD

Model comparison and random effect analysis

Table 3 illustrates that model III in the multilevel analysis outperforms all other multilevel models and the standard logistic regression model (i.e., the model that included all variables but did not include random effect), as it has the highest log likelihood (-1414.7885) and the lowest deviance (2,830) value. According to the ICC value in the null model, cluster differences were responsible for nearly 14.45% of the variation in MDD among children. The MOR between the higher and lower areas among clusters was 2.03, as shown by the MOR in the null model. Furthermore, both individual and community-level variables accounted for around 21% of the heterogeneity in MDD among children [Table 3].

Fixed-effect analysis

In the final fitted model of multilevel logistic regression analysis (model III), the following factors, like media exposure, household wealth index, ANC visits, current breastfeeding status, age of the child, number of under-5 children in the household, and community-level media exposure, were

significantly associated with MDD. Accordingly, the odds of meeting MDD were 1.51 times higher among mothers who were exposed to media compared with those who weren't [AOR = 1.51; 95% CI (1.08, 2.13)]. Mothers with middle and rich wealth status were 1.58 and 1.84 times more likely to meet MDD than those with poor wealth status, respectively [AOR = 1.58; 95% CI (1.12, 2.24)] and [AOR = 1.84; 95% CI (1.24, 2.74)]. Mothers who attended four or more ANC visits were 1.50 times more likely to meet MDD than those who didn't attend ANC visits [AOR = 1.50; 95% CI (1.05, 2.13)]. Breastfeeding children were 1.82 times more likely to achieve MDD compared with their counterparts [AOR = 1.82; 95% CI (1.37, 2.43)]. Children aged 12–17 months and 18–23 months were 5.21 and 7.47 times more likely to achieve MDD than those aged 6–11 months, respectively [AOR = 5.21; 95% CI (3.94, 6.87)] and [AOR = 7.47; 95% CI (5.43, 10.3)]. Mothers who had no under-five children in the household were 3.97 times more likely to meet MDD than those who had three or more under-five children [AOR = 3.97; 95% CI (1.38, 11.4)]. Mothers from communities with high levels of media exposure were 1.41 times more likely to meet MDD compared with their counterparts [AOR = 1.41; 95% CI (1.03, 1.93)] [Table 4].

Discussion

The long-term burden of stunting among newborns and young children can be significantly decreased by interventions aimed at improving dietary diversity, which determines the nutritional status of children and is a key predictor of stunting (33, 34). In the present study, the proportion of adequate MDD among children aged 6–23 months in Senegal was 23.61% (95% CI: 22.12, 25.16). This finding is consistent with studies conducted in India (22.46%) (35), 32 sub-Saharan African countries (25.1%) (13), and three sub-Saharan African countries (23.2%) (21). On the other hand, the current finding is higher than studies conducted in Ethiopia (13.4% & 14.9%) (22, 36) and Pakistan (20%) (24). However, this finding is lower than studies conducted in the Gedeo zone,

Ethiopia (29.9%) (25), and Bangladesh (38%) (23). The possible justification for this similarity and discrepancy can be explained by variations in geographical location, study period, seasonal variability, cultural variation, sample size, and other socioeconomic factors.

The study also identified individual and community-level factors significantly associated with MDD. Accordingly, the odds of meeting MDD were higher among mothers who were exposed to media compared with those who weren't. Likewise, mothers from communities with high levels of media exposure were more likely to meet MDD. This finding is in agreement with studies conducted in Ethiopia (22, 36) and three sub-Saharan African countries (21). The media that women are exposed to, such as reading newspapers, watching TV, and listening to the radio, can encourage them to use maternal healthcare facilities (37, 38). Mass media's informative, instrumental, social control, and community roles in health communication might influence attitudes toward changes in healthy behavior (39). Children from wealthier households were more likely to achieve MDD. This finding is consistent with studies conducted in Ethiopia (25, 36), three sub-Saharan African countries (21), and Bangladesh (23). This may be explained by the high cost of a variety of foods and the idea that eating them is a luxury. Rather than providing their children with a variety of foods, mothers from low-income backgrounds may also sell their animals and goods in order to get enough money to pay for their children's education, food, and other needs.

Mothers who attended four or more ANC visits were more likely to meet MDD than those who didn't. This finding is in line with studies conducted in Indonesia (26) and South Asia (27). This may be the result of mothers' increased awareness of suitable child feeding practices brought about by the information and counseling they receive from health care providers while using ANC services. This suggests that in order to enhance the eating behaviors of infants and young children, the usage of maternity services should be encouraged. Breastfeeding children were more likely to

achieve MDD compared with their counterparts. This finding is in agreement with a study conducted in Brazil (40). A plausible explanation for the correlation between extended breastfeeding and improved feeding practices in later childhood is that families who adhere to the advice to continue breastfeeding for a minimum of two years without providing non-human milk are also likely to prioritize other dietary recommendations for a healthy lifestyle (41).

Likewise, older children were more likely to achieve MDD than younger ones. This finding is consistent with studies conducted in the Gedeo zone, Ethiopia (25), three sub-Saharan African countries (21), and Pakistan (24). The fact that older children are more accustomed to food than younger children and are more willing to consume a variety of foods with varying tastes and textures could be one explanation for this link (26). In addition, this outcome may be the result of mothers generally delaying the introduction of cereals and legumes to their very young children (6–11 months) until the child turns one year old (42). Mothers who had no under-five children in the household were more likely to meet MDD than those who had three or more under-five children. This finding is in line with a study conducted in Ethiopia (43). One explanation could be because mothers with large numbers of under five children are less likely to buy a variety of food categories and are therefore unable to provide for their children's nutritional needs.

Strengths and limitations of the study

The main strength of the study is utilizing weighted nationally representative data with a large sample size, which makes it representative at the national level. The present study also has sufficient statistical power to generalize the estimates of MDD among children aged 6 to 23 months during the research period. On the other hand, recall and social desirability bias are likely to arise due to the cross-sectional nature of the data collection method (self-reported). The secondary nature of data was also another limitation of this study, because key variables that affect MDD,

like local agriculture, distance to major cities, seasonality, and local climate, were not incorporated in the current analysis.

Conclusion

Nearly one in four children aged 6 to 23 months achieved minimum dietary diversity in Senegal. Children from mothers with media exposure, wealthier households, who attended 4+ ANC visits, who are currently breastfeeding, who are older children, and who have no under-5 children in the household were associated with higher odds of meeting MDD. Therefore, improving media access, women's empowerment, enhancing ANC service utilization, encouraging continued breastfeeding, and giving prior attention to younger children aged 6 to 11 months and mothers who had under-five children in the household are strongly recommended to improve feeding practices of infants and young children.

List of abbreviations: ANC: Antenatal Care; AOR: Adjusted Odds Ratio; CI: Confidence Interval; DHS: Demographic and Health Survey; ICC: Intra-class Correlation Coefficient; MDD: Minimum Dietary Diversity; MOR: Median Odds Ratio; PCV: Proportional Change in Variance; VIF: Variance Inflation Factor; WHO: World Health Organization

Consent for publication: Not applicable.

Availability of data and materials: The data from the DHS is publicly available online at https://dhsprogram.com/data/available-datasets.cfm.

Competing interest: The author declared that there was no competing interest.

Funding: Not applicable

Author contributions: Conceptualization, data curation, formal analysis, investigation, methodology, software, visualization, writing the original draft, reviewing, and editing were done by E.G.M.

Acknowledgments: I am grateful to the DHS program for letting me use the relevant data in this study.

References

1. UNICEF. Early childhood nutrition; Preventing malnutrition in infants and young children. 2024.

2. UNICEF. Improving young children's diets during the complementary feeding period. UNICEF programming guidance. 2020;76:118-22.2023.

3. Dewey K. Guiding principles for complementary feeding of the breastfed child. 2003.

4. WHO. Guiding principles for feeding non-breastfed children 6-24 months of age: World Health Organization; 2005.

5. Onyango AW, Borghi E, de Onis M, del Carmen Casanovas M, Garza C. Complementary feeding and attained linear growth among 6–23-month-old children. Public health nutrition. 2014;17(9):1975-83.

6. Prado EL, Dewey KG. Nutrition and brain development in early life. Nutrition reviews. 2014;72(4):267-84.

7. Aguayo VM, Nair R, Badgaiyan N, Krishna V. Determinants of stunting and poor linear growth in children under 2 years of age in India: An in-depth analysis of Maharashtra's comprehensive nutrition survey. Maternal & child nutrition. 2016;12:121-40.

8. Leal KK, Schneider BC, França GVA, Gigante DP, Santos Id, Assunção MCF. Diet quality of preschool children aged 2 to 5 years living in the urban area of Pelotas, Brazil. Revista Paulista de Pediatria. 2015;33:310-7.

9. Zhou Z, Liew J, Yeh Y-C, Perez M. Appetitive traits and weight in children: evidence for parents' controlling feeding practices as mediating mechanisms. The Journal of genetic psychology. 2020;181(1):1-13.

10. Lutter CK, Grummer-Strawn L, Rogers L. Complementary feeding of infants and young children 6 to 23 months of age. Nutrition Reviews. 2021;79(8):825-46.
11. Bhandari N. Infant and young child feeding. 2016.
12. WHO. Indicators for assessing infant and young child feeding practices: definitions and measurement methods. 2021.
13. Aboagye RG, Seidu A-A, Ahinkorah BO, Arthur-Holmes F, Cadri A, Dadzie LK, et al. Dietary diversity and undernutrition in children aged 6–23 months in Sub-Saharan Africa. Nutrients. 2021;13(10):3431.
14. Kennedy GL, Pedro MR, Seghieri C, Nantel G, Brouwer I. Dietary Diversity Score Is a Useful Indicator of Micronutrient Intake in Non-Breast-Feeding Filipino Children2. The Journal of nutrition. 2007;137(2):472-7.
15. McDonald CM, Olofin I, Flaxman S, Fawzi WW, Spiegelman D, Caulfield LE, et al. The effect of multiple anthropometric deficits on child mortality: meta-analysis of individual data in 10 prospective studies from developing countries. The American journal of clinical nutrition. 2013;97(4):896-901.
16. Khamis AG, Mwanri AW, Ntwenya JE, Kreppel K. The influence of dietary diversity on the nutritional status of children between 6 and 23 months of age in Tanzania. BMC pediatrics. 2019;19:1-9.
17. Saha J, Chouhan P, Malik NI, Ghosh T, Das P, Shahid M, et al. Effects of Dietary Diversity on Growth Outcomes of Children Aged 6 to 23 Months in India: Evidence from National Family and Health Survey. Nutrients. 2022;15(1):159.
18. Ballini L. Food Insecurity and Lack of Dietary Diversity: Its Association with Anemia In Senegalese Women and Children.
19. Janmohamed A, Baker MM, Doledec D, Ndiaye F, Konan ACL, Leonce A, et al. Dietary diversity and associated factors among infants and young children in three West African countries. Frontiers in Public Health. 2024;12:1386664.
20. POUYE R, Ndicak F. Nutritional status and associated factors in children under five in Senegal: an empirical analysis. Moroccan Journal of Public Heath. 2023;5(1).
21. Ba DM, Ssentongo P, Gao X, Chinchilli VM, Richie Jr JP, Maiga M, et al. Prevalence and determinants of meeting minimum dietary diversity among children aged 6–23 months in three

sub-Saharan African Countries: The Demographic and Health Surveys, 2019–2020. Frontiers in Public Health. 2022;10:846049.

22. Eshete T, Kumera G, Bazezew Y, Mihretie A, Marie T. Determinants of inadequate minimum dietary diversity among children aged 6–23 months in Ethiopia: secondary data analysis from Ethiopian Demographic and Health Survey 2016. Agriculture & Food Security. 2018;7(1):1-8.

23. Khanam M, Sarker AR. Dietary Diversity among Children Aged 6-23 Months in Bangladesh. The Bangladesh Development Studies. 2021;44(3/4):81-102.

24. Kumar R, Mahmood T, Naeem N, Khan SA, Hanif M, Pongpanich S. Minimum dietary diversity and associated determinants among children aged 6–23 months in Pakistan. Scientific Reports. 2024;14(1):2639.

25. Molla W, Adem DA, Tilahun R, Shumye S, Kabthymer RH, Kebede D, et al. Dietary diversity and associated factors among children (6–23 months) in Gedeo zone, Ethiopia: cross-sectional study. Italian journal of pediatrics. 2021;47(1):233.

26. Paramashanti BA, Huda TM, Alam A, Dibley MJ. Trends and determinants of minimum dietary diversity among children aged 6–23 months: a pooled analysis of Indonesia demographic and health surveys from 2007 to 2017. Public health nutrition. 2022;25(7):1956-67.

27. Rahman MA, Kundu S, Rashid HO, Tohan MM, Islam MA. Socio-economic inequalities in and factors associated with minimum dietary diversity among children aged 6–23 months in South Asia: a decomposition analysis. BMJ open. 2023;13(12):e072775.

28. National Agency for Statistics and Demography (NASD) [Senegal] aI. Demographic Survey and Continuing Health (2023 Continuous Survey) Maryland, USA: NASD and ICF. 2023.

29. Macro O. Demographic and health surveys. Calverton, MD: ICF Macro. 2004.

30. Croft TN, Allen, Courtney K., Zachary, Blake W., et al. Guide to DHS Statistics. Rockville, Maryland, USA: ICF. 2023.

31. Daoud JI, editor Multicollinearity and regression analysis. Journal of Physics: Conference Series; 2017: IOP Publishing.

32. Sommet N, Morselli D. Keep calm and learn multilevel logistic modeling: A simplified three-step procedure using Stata, R, Mplus, and SPSS. International Review of Social Psychology. 2017;30:203-18.

33. Ahmad I, Khalique N, Khalil S, Maroof M. Dietary diversity and stunting among infants and young children: a cross-sectional study in Aligarh. Indian Journal of Community Medicine. 2018;43(1):34-6.
34. Borkotoky K, Unisa S, Gupta AK. State-level dietary diversity as a contextual determinant of nutritional status of children in India: a multilevel approach. Journal of biosocial science. 2018;50(1):26-52.
35. Padigapati VNS, Singh A, KV P. Are children attaining the minimum dietary diversity criteria in India? Evidence from National Family Health Survey-5. 2024.
36. Mulaw GF, Mohammed OA, Mare KU. Level of dietary diversity score and its predictors among children aged 6–23 months: a linear mixed model analysis of the 2019 Ethiopian Mini Demographic Health Survey. BMJ Public Health. 2024;2(1).
37. Aboagye RG, Seidu A-A, Ahinkorah BO, Cadri A, Frimpong JB, Hagan JE, et al. Association between frequency of mass media exposure and maternal health care service utilization among women in sub-Saharan Africa: implications for tailored health communication and education. Plos one. 2022;17(9):e0275202.
38. Fatema K, Lariscy JT. Mass media exposure and maternal healthcare utilization in South Asia. SSM-Population Health. 2020;11:100614.
39. Sarrassat S, Meda N, Ouedraogo M, Some H, Bambara R, Head R, et al. Behavior change after 20 months of a radio campaign addressing key lifesaving family behaviors for child survival: midline results from a cluster randomized trial in rural Burkina Faso. Global Health: Science and Practice. 2015;3(4):557-76.
40. Bortolini GA, Giugliani ERJ, Gubert MB, Santos LMP. Breastfeeding is associated with children's dietary diversity in Brazil. Ciência & Saúde Coletiva. 2019;24:4345-54.
41. Vitolo MR, Bortolini GA, Campagnolo PDB, Hoffman DJ. Maternal dietary counseling reduces consumption of energy-dense foods among infants: a randomized controlled trial. Journal of nutrition education and behavior. 2012;44(2):140-7.
42. Baek Y, Chitekwe S. Sociodemographic factors associated with inadequate food group consumption and dietary diversity among infants and young children in Nepal. PloS one. 2019;14(3):e0213610.
43. Woldegebriel AG, Desta AA, Gebreegziabiher G, Berhe AA, Ajemu KF, Woldearegay TW. Dietary Diversity and Associated Factors among Children Aged 6-59 Months in Ethiopia: Analysis of Ethiopian Demographic and Health Survey 2016 (EDHS 2016). International journal of pediatrics. 2020;2020(1):3040845.

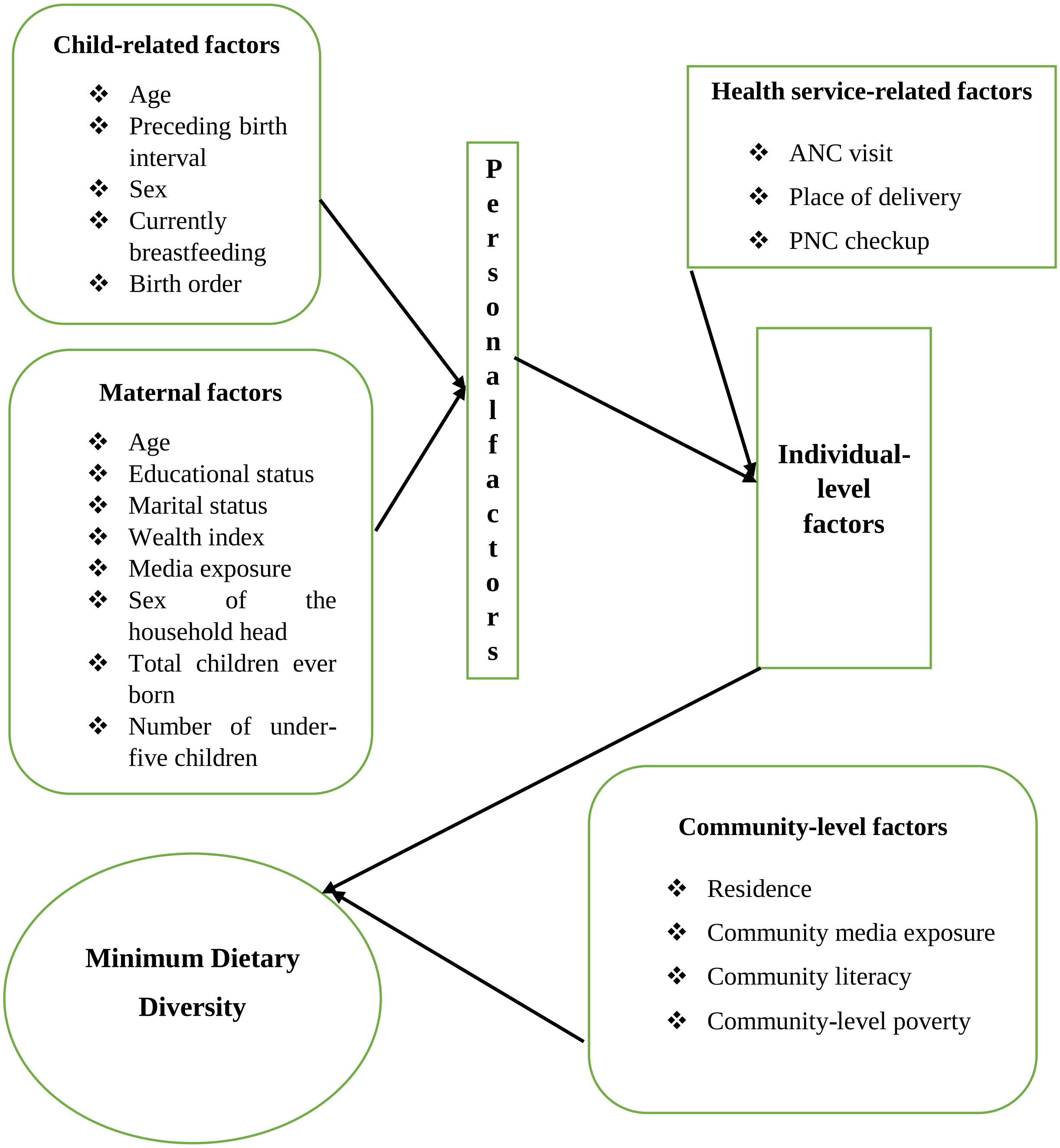

Figure 1: Conceptual framework for factors associated with minimum dietary diversity among children aged 6 to 23 months in Senegal

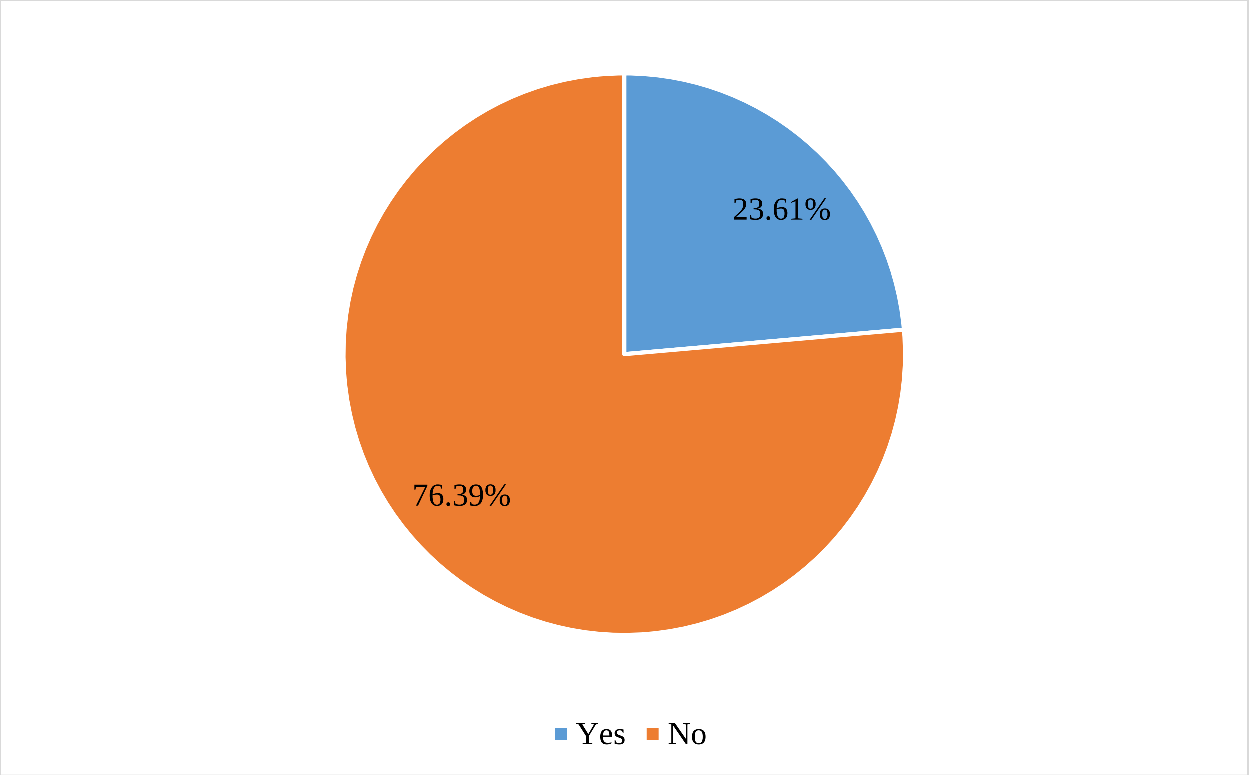

Figure 2: Proportion of children aged 6-23 months who meet minimum dietary diversity in Senegal; DHS 2023 (n = 2,995)

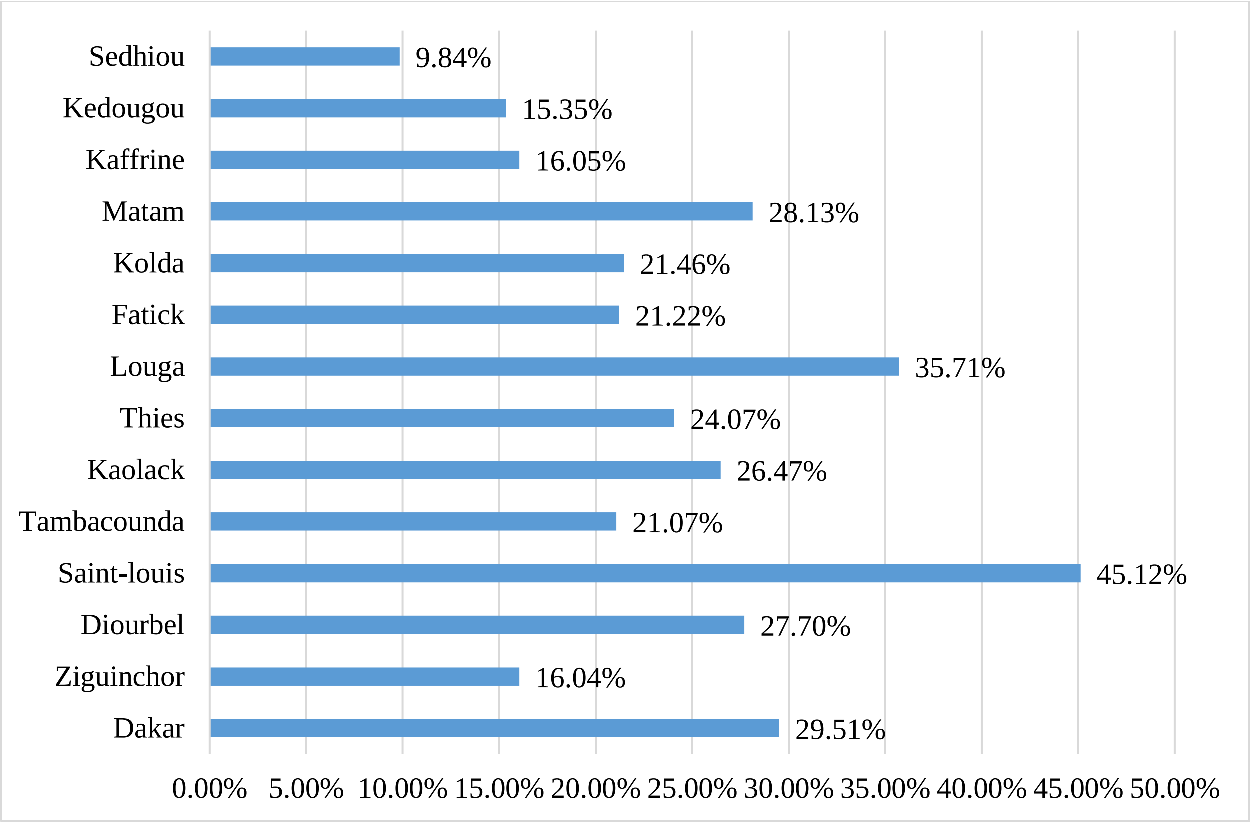

Figure 3: Regional proportion of children aged 6-23 months who meet minimum dietary diversity in Senegal; DHS 2023 (n = 2,995)

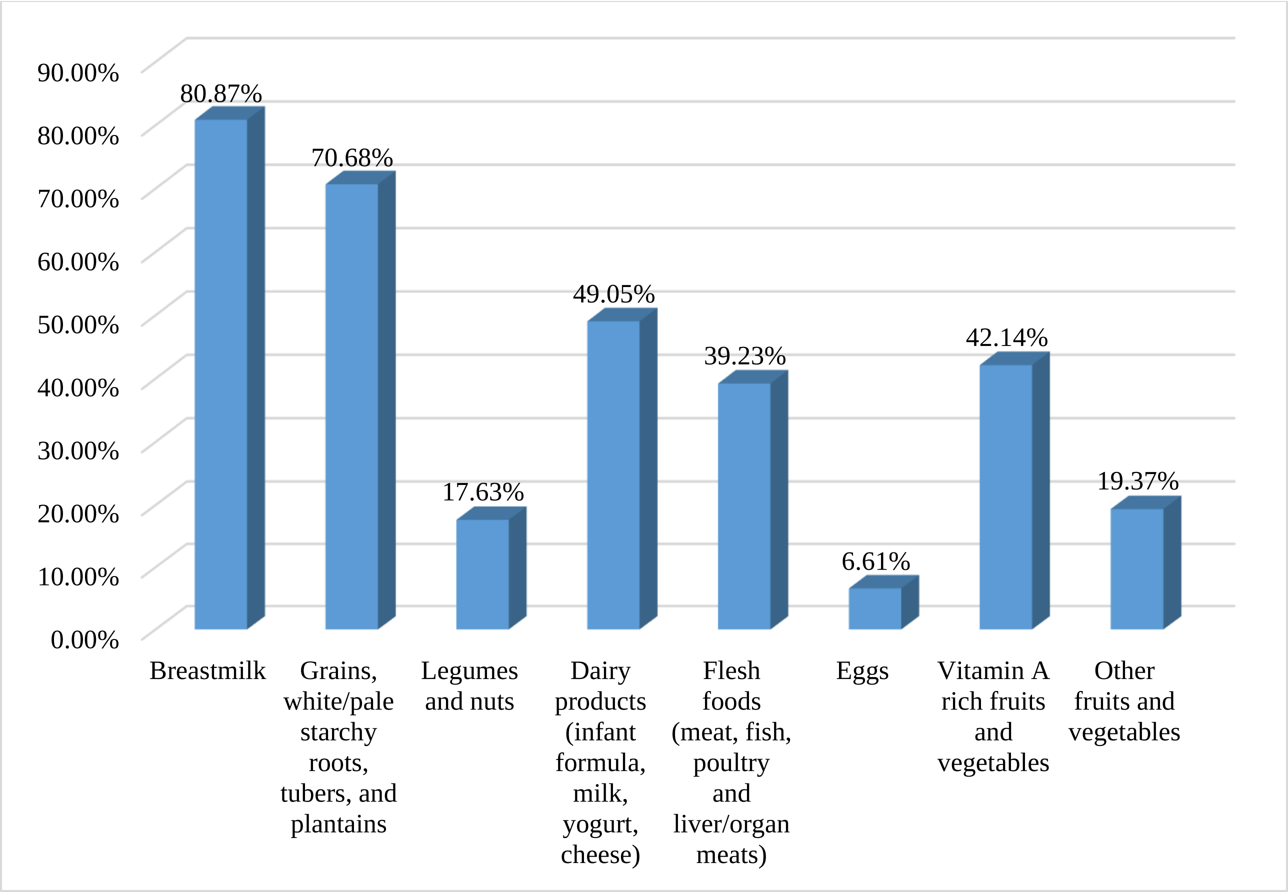

Figure 4: Proportion of children aged 6-23 months who consumed the eight defined food groups in Senegal; DHS 2023 (n = 2,995)

Table 1: Individual-and community-level characteristics of study subjects in Senegal; DHS 2023

Variables	Category	Frequency (n)	Percentage (%)
Maternal age	15–24 years	1,083	36.16
	25–34 years	1,266	42.27
	35–49 years	646	21.57
Maternal education	No education	1,641	54.79
	Primary	564	18.83
	Secondary & higher	790	26.38
Marital status	Unmarried	165	5.51
	Married	2,830	94.49
Sex of the household head	Male	2,376	79.33
	Female	619	20.67
Total children ever born	1-3	1,829	61.07
	4-6	849	28.35
	≥7	317	10.58
Media exposure	No	522	17.43
	Yes	2,473	82.57
Wealth index	Poor	1,732	57.83
	Middle	562	18.76
	Rich	701	23.41
ANC visits	None	295	9.85
	1-3 visits	666	22.24
	4+ visits	2,034	67.91
Place of delivery	Home	284	9.48
	Health facility	2,711	90.52
Currently breastfeeding	No	540	18.03
	Yes	2,455	81.97
PNC checkup	No	1,677	57.77
	Yes	1,226	42.23
Sex of the child	Male	1,545	51.59
	Female	1,450	48.41

Age of the child	6–11 months	982	32.79
	12–17 months	1,141	38.09
	18–23 months	872	29.12
Birth order	First-born	735	24.54
	2-4	1,466	48.95
	5+	794	26.51
Preceding birth interval	< 24 months	18	0.60
	≥ 24 months	2,977	99.40
Under-5 children in the household	None	20	0.67
	1–2	1,306	43.60
	3+	1,669	55.73
Place of residence	Rural	1,934	64.57
	Urban	1,061	35.43
Community literacy	Low	1,516	50.62
	High	1,479	49.38
Community poverty level	low	1,652	55.16
	High	1,343	44.84
Community media exposure	Low	1,001	33.42
	High	1,994	66.58

Table 2: Patterns and differentials of MDD by background characteristics

**Independent variables**		**Meeting MDD**	
Media exposure	No	No	Yes
		19.62%	10.32%
	Yes	80.38%	89.68%
Wealth index	Poor	61.41%	46.25%
	Middle	17.66%	22.35%
	Rich	20.93%	31.40%
ANC visits	None	10.36%	8.20%
	1-3	23.38%	18.53%
	4+	66.26%	73.27%
Currently breastfeeding	No	17.40%	20.08%
	Yes	82.60%	79.92%
Child age	6-11 months	39.12%	12.31%
	12-17 months	34.97%	48.23%
	18-23 months	25.91%	39.46%
Number of under-five children	None	0.48%	1.27%
	1-2	42.66%	46.68%
	3+	56.86%	52.05%
Community media exposure	Low	36.54%	23.34%
	High	63.46%	76.66%

Table 3: Model comparison and random effect analysis for minimum dietary diversity and its associated factors among women in Senegal; DHS 2023 (n = 2,995)

Parameter	Null model	Model I	Model II	Model III
Variance	0.555578	0.4460063	0.4114763	0.437621
ICC	14.45%	11.94%	11.12%	11.74%
MOR	2.03	1.88	1.84	1.87
PCV	Reference	19.72%	25.94%	21.23%
**Model fitness**				
LLR	-1608.4565	-1417.4869	-1588.1932	-1414.7885
Deviance	3,216.913	2,834.9738	3,176.3864	2,829.577

ICC: Intra cluster correlation; LLR: Log-likelihood ratio; MOR: Median odds ratio; PCV: Proportional change in variance

Table 4: Multivariable multilevel logistic regression analysis of factors associated with minimum dietary diversity among children in Senegal; DHS 2023 (n = 2,995)

Variables	Category	Model I AOR (95% CI)	Model II AOR (95% CI)	Model III AOR (95% CI)
Maternal age	15–24 years	1.00		1.00
	25–34 years	1.05 (0.81, 1.37)		1.05 (0.81, 1.37)
	35–49 years	0.89 (0.62, 1.30)		0.89 (0.62, 1.30)
Maternal education	No education	1.00		1.00
	Primary	0.92 (0.70, 1.20)		0.91 (0.69, 1.19)
	Secondary & higher	0.85 (0.65, 1.11)		0.83 (0.63, 1.11)
Marital status	Unmarried	1.00		1.00
	Married	1.18 (0.74, 1.87)		1.19 (0.75, 1.89)
Sex of the household head	Male	1.00		1.00
	Female	1.09 (0.86, 1.39)		1.08 (0.85, 1.38)
Total children ever born	1-3	0.76 (0.44, 1.31)		0.76 (0.44, 1.31)
	4-6	0.75 (0.50, 1.13)		0.75 (0.50, 1.13)
	≥7	1.00		1.00
Media exposure	No	1.00		1.00
	Yes	1.74(1.26, 2.39)*		1.51(1.08, 2.13)*
Wealth index	Poor	1.00		1.00
	Middle	1.67(1.27, 2.21)*		1.58(1.12, 2.24)*
	Rich	2.01(1.51, 2.69)*		1.84(1.24, 2.74)*
ANC visits	None	1.00		1.00
	1-3 visits	1.18 (0.79, 1.76)		1.19 (0.80, 1.77)
	4+ visits	1.50(1.05, 2.13)*		1.50(1.05, 2.13)*
Place of delivery	Home	1.00		1.00
	Health facility	1.16 (0.79, 1.71)		1.13 (0.77, 1.66)
Currently breastfeeding	No	1.00		1.00
	Yes	1.82(1.37, 2.43)*		1.82(1.37, 2.43)*
PNC checkup	No	1.00		1.00
	Yes	1.07 (0.87, 1.31)		1.07 (0.87, 1.31)

Sex of the child	Male	1.00		1.00
	Female	1.05 (0.87, 1.28)		1.05 (0.87, 1.28)
Age of the child	6–11 months	1.00		1.00
	12–17 months	5.21(3.95, 6.88)*		5.21(3.94, 6.87)*
	18–23 months	7.46(5.43, 10.3)*		7.47(5.43, 10.3)*
Birth order	First-born	0.86 (0.54, 1.38)		0.86 (0.54, 1.38)
	2-4	0.89 (0.62, 1.29)		0.89 (0.62, 1.29)
	5+	1.00		1.00
Preceding birth interval	< 24 months	1.00		1.00
	≥ 24 months	0.61 (0.19, 2.03)		0.61 (0.18, 2.03)
Under-5 children in the household	None	4.13(1.44, 11.8)*		3.97(1.38, 11.4)*
	1–2	1.17 (0.96, 1.44)		1.17 (0.95, 1.44)
	3+	1.00		1.00
Place of residence	Rural		1.00	1.00
	Urban		1.24(0.91, 1.68)	1.10 (0.79, 1.53)
Community literacy	Low		1.00	1.00
	High		1.00(0.78, 1.28)	1.06 (0.80, 1.40)
Community poverty level	low		0.77(0.56, 1.06)	1.09 (0.72, 1.63)
	High		1.00	1.00
Community media exposure	Low		1.00	1.00
	High		1.67(1.26,2.20)*	1.41(1.03, 1.93)*

*Statistically significant at P-value<0.05; ANC: Antenatal care; PNC: Postnatal care

Click here to access/download
Supporting Information
PLOSOne_Human_Subjects_Research_Checklist.docx

Minimum Dietary Diversity and its Associated Factors among Children Aged 6–23 Months in Senegal: Evidence from the Demographic and Health Survey, 2023

Enyew Getaneh Mekonen

Department of Surgical Nursing, School of Nursing, College of Medicine and Health Sciences, University of Gondar, Gondar, Ethiopia

enyewgetaneh12@gmail.com

Abstract

Background: Children between the ages of 6 and 23 months should be provided a variety of foods to ensure that their nutrient needs are satisfied, according to World Health Organization guidelines for feeding children. In Senegal, lower dietary diversity and an inability to meet nutrient requirements raise the risk of anemia and other disorders caused by a lack of funds and resources, which are essential for consuming a nutrient-rich diet. The objective of this study was to assess minimum dietary diversity and its associated factors among children aged 6–23 months in Senegal using the most recent demographic and health survey data (2023).

Methods: A cross-sectional study was conducted in Senegal using data from the most recent continuous demographic and health survey, 2023. A total of 2,995 children aged 6–23 months were included in the study. Using STATA Version 14 software, the data were extracted, cleaned, and analyzed. A multilevel mixed-effects logistic regression analysis was used to determine the factors associated with meeting minimum dietary diversity~~the outcome variable~~. Finally, variables with a p-value less than 0.05 were declared statistically significant.

Use italic for p in p-value

Results: In the present study, the proportion of children aged 6-23 months who meet minimum dietary diversity in Senegal was 23.61% (95% CI: 22.12, 25.16). Factors like media exposure [AOR = 1.51; 95% CI (1.08, 2.13)], household wealth index [AOR = 1.84; 95% CI (1.24, 2.74)], ANC visits [AOR = 1.50; 95% CI (1.05, 2.13)], current breastfeeding status [AOR = 1.82; 95% CI (1.37, 2.43)], age of the child [AOR = 7.47; 95% CI (5.43, 10.3)], number of under-5 children in the household [AOR = 3.97; 95% CI (1.38, 11.4)], and community-level media exposure [AOR = 1.41; 95% CI (1.03, 1.93)] were significantly associated with meeting minimum dietary diversity.

Conclusions: Nearly one in four children aged 6 to 23 months achieved minimum dietary diversity in Senegal. Children from mothers with media exposure, wealthier households, attending 4+ antenatal care visits, currently breastfeeding, older children, and no under-5 children in the household were associated with higher odds of meeting minimum dietary diversity. Therefore, improving media access, women empowerment, enhancing antenatal care service utilization, encouraging continued breastfeeding, and giving prior attention to younger children aged 6 to 11 months and mothers who had under-five children in the household are strongly recommended.

Keywords: minimum dietary diversity, DHS, multilevel analysis, young children, Senegal

Background

For children to grow and develop to the best extent possible, the proper meals must be consumed at the right times. When it comes to nutrition, the 1,000 days from conception to the child's second birthday are the most important (1). Children need to start eating their first foods around six months of age. Throughout the day, young children should be fed regularly, in sufficient amounts, and with a range of food groups in their meals that are high in nutrients. In addition to using clean

hands and dishes during meal preparation and serving, caregivers should engage with their children to acknowledge their ~~hunger cues~~ nutritional requirement (2).

Children between the ages of 6 and 23 months should be provided a variety of foods to ensure that their nutrient needs are satisfied, according to World Health Organization (WHO) guidelines for feeding breastfed and non-breastfed children (3, 4). Variety in food groups is linked to better linear growth in early children (5). Micronutrient deficiencies may be more likely in a diet low in diversity, which could be detrimental to children's physical and mental development (6). According to a previous study, stunting was associated with little to no ingestion of nutrient-dense foods such as eggs, dairy products, fruits, and vegetables between the ages of six and 23 months (7).

Feeding infants and young children involves more than just making sure they get enough nourishment. Meals are social and cultural gatherings when young children learn about food preferences and dislikes, observe, and mimic behavior that will shape their eating habits for the rest of their lives (8). A child learns to handle food during meals and makes the connection between food tastes, appearances, and textures. Early child feeding should ideally encourage a child's independence, but it can also be used to control behavioral issues or give a child too much, which can have long-term negative effects on the child's nutrition and health (9). It's crucial to pay attention to how a child is fed in addition to what they eat (10).

To make it practical and relevant for Member State reporting, the WHO-UNICEF Technical Expert Advisory Group on Nutrition Monitoring (TEAM) suggested in June 2017 revising the minimum dietary diversity (MDD) indicator as defined by WHO (2008). The adjustment involved moving the MDD criterion from four of seven categories to five of eight groups, and adding "breast milk" as an eighth dietary group (11). Therefore, if a child consumes five or more of the following

food groups, their diet is considered diverse: breast milk; grains, roots, and tubers; legumes and nuts; dairy products (milk, yogurt, cheese); flesh foods (meat, fish, poultry, liver, or other organs); eggs; vitamin A-rich fruits and vegetables; and other fruits and vegetables (12).

The relationship between dietary diversity and children's health is important, as it is a protective factor against low nutritional status, which can show up as underweight, which is a combination of stunting and wasting in children; wasting, which is an acute form of undernutrition; and stunting, which is chronic and long-term undernutrition (13-15). A diverse diet is linked to a decrease in undernutrition in children between the ages of six and twenty-three months (16). It was also observed that children who abstained from eating eggs, dairy products, and other fruits and vegetables had a higher probability of being underweight, severely stunted, and wasted (17).

Still needs to address the general research problem, i.e. that in Senegal the negative impact of inadequate minimum dietary diversity, such as lower education achievement, is a problem, usually addressed in the national development plan.

In Senegal, lower dietary diversity and an inability to meet nutrient requirements raise the risk of anemia and other disorders caused by a lack of funds and resources, which are essential for consuming a nutrient-rich diet (18). The prevalence of MDD was 30.8% in Senegal, and the socioeconomic barriers to achieving dietary diversity were identified (19). The high prevalence of micronutrient malnutrition in Senegal makes it imperative to promptly analyze the nutritional adequacy of young children's meals in order to target the most vulnerable groups with suitable interventions (20). Studies conducted elsewhere showed that media exposure, household wealth index, age of the child, maternal education, number of families, number of under-five children, ANC visits, and residence were determinants of meeting MDD (19, 21-27). ~~Therefore, t~~The objective of this study was to assess minimum dietary diversity and its associated factors among children aged 6–23 months in Senegal using the most recent demographic and health survey (DHS) data (2023).

Still needs to address the novelty of this study.

Methods and materials

Data sources, study design, and setting

Cross-sectional data from the Continuous DHS in Senegal in 2023 was used for the present study. The DHS-C 2023 is the fifth edition after the continuous surveys conducted in Senegal between 2012–2014, 2015–2016, 2017–2018, and 2019. Other DHS surveys were conducted in Senegal in 1986, 1992–93, 1997, 2005, and 2010. This edition of the survey used a nationally representative sample of 400 clusters and 8,800 households, with an expected number of 16,142 women aged 15-49 surveyed with success. All women aged 15 to 49 who are members of households or who have spent the night before the day of the survey in selected households are eligible for the survey. As with the previous DHS~~EDS surveys~~, the main purpose of the DHS-C 2023 ~~ESDC 2023 survey~~ is to gather information on the health of women and their young children, on fertility, on the knowledge and use of contraceptive methods, and on knowledge and attitudes towards sexually transmitted diseases. The results of the survey are presentative for Senegal, for the urban environment and rural areas separately, and for each of the fourteen administrative regions. The National Agency for Statistics and Demography (NASD) has a computer file of Census Districts (DRs) created as a result of the General Population and Housing Census, Agriculture, and Livestock of 2013 (RGPHAE-2013). The datasets are publicly available from the DHS website, www.dhsprogram.com. The surveys are nationally representative of the country and population-based with large sample sizes (28).

Source and study populations

The youngest child, age 6–23 months, living with the mother in Senegal comprised the source population, whereas women in the selected primary sampling unit (PSU) comprised the study

population. Weighted values, which were computed from the Kid's record (KR file) Senegal DHS 2023 datasets, were utilized to restore the representativeness of the sample data. Finally, this study comprised a weighted sample of 2,995 children aged 6-23 months.

Sampling procedure

The 2023 DHS-C sample is a random, stratified sample drawn at 2 degrees. The primary unit of survey is the DR as defined for the 2013 Census. Each region was separated into parts to form the sampling strata, and the sample was drawn independently from each sampling layer. A total of 28 sample strata were created. Inside each stratum, the DRs were sorted by administrative units below the region, i.e., departments and districts/communes, etc. This operation had introduced implicit stratification at the level of all administrative units below the region with an implicit stratification of the sample allocation proportional to unit size. Prior to the enumeration of households, each large DR having more than 200 households was divided into segments, of which only one was included in the sample. At the second level, in each of the DRs selected in the first, a fixed number of 22 households had been selected with a systematic probability draw from the newly established lists at the time of enumeration. Only the households drawn are interviewed. Replacements of pre-selected households were not allowed immediately, even if they were not for non-responding households, to avoid bias. Every DHS report could be accessed on the Measure DHS website, which included the comprehensive sampling procedure (29).

Variables of the study

Dependent variable

The outcome variable of this study was the minimum dietary diversity status of the child (adequate/inadequate). Children aged 6–23 months who consumed foods and beverages five or

more out of the eight defined food groups during the day or night before the survey were considered as meeting MDD (adequate), and inadequate otherwise.~~The outcome variable of this study was minimum dietary diversity (adequate/inadequate) defined as the proportion of children aged 6–23 months who consumed foods and beverages five or more out of the eight defined food groups during the day or night before the survey~~. The eight food groups are a) breastmilk, b) grains, white/pale starchy roots, tubers, and plantains, c) legumes and nuts, d) dairy products (infant formula, milk, yogurt, cheese), e) flesh foods (meat, fish, poultry, and liver/organ meats), f) eggs, g) vitamin A-rich fruits and vegetables, and h) other fruits and vegetables (30).

Independent variables

Independent variables from two sources (variables at the individual and community levels) were taken into consideration for this analysis, as DHS data are hierarchical in nature. Variables included at the individual-level were maternal age (15-24, 25-34, 35-49 years), maternal education (no education, primary, secondary & higher), marital status (unmarried, married), sex of the household head (male, female), total children ever born (1-3, 4-6, ≥7), media exposure (no, yes), wealth index (poor, middle, rich), ANC visits (none, 1-3 visits, 4+ visits), place of delivery (home, health facility), currently breastfeeding (no, yes), PNC checkup (no, yes), sex of the child (male, female), age of the child (6 – 11, 12 – 17, 18 – 23 months), birth order (first-born, 2-4, 5+), preceding birth interval (< 24 months, ≥ 24 months), and the number of under-5 children in the household (none, 1–2, 3+). Variables included at the community-level were place of residence (urban, rural), community literacy (low, high), community-level poverty (low, high), and community media exposure (low, high) (Figure 1).

Operational description of independent variables

Media exposure was established by combining three factors: reading newspapers, watching television, and listening to the radio. A woman was deemed to have been exposed to media if she had at least one kind of exposure.

Media exposure at the community level was measured using the percentage of women who had at least been exposed to one of the three media: television, radio, or newspapers. It was coded as "0" for low community-level media exposure (communities where less than 50% of women had at least one media exposure) and "1" for high community-level media exposure (communities where more than 50% of women had at least one media exposure).

The level of poverty in the community was calculated based on the percentage of women in the lowest and poorest quintiles, as obtained from the wealth index data. The poverty levels were classified as "0" for low (those where less than 50% of women belonged to the lowest and worst wealth quintiles) and "1" for high (those where more than 50% of women belonged to the poorest and poorer wealth quintiles) communities.

Women's literacy at the community level was also measured using the percentage of women with at least a primary education. It was coded as "0" for low community-level women's education (communities where less than 50% of women had completed primary school) and "1" for high community-level women's education (communities where more than 50% of women completed primary education at the cluster level). Is there any statistical problem using maternal education and this community problem in the same model?

Data collection tools and quality assurance

Demographics and health surveys use interviewer-administered questionnaire methodologies to gather data through several questionnaire formats. The DHS guideline defined the missing values in the outcome variables in a straightforward manner (30). However, as complete case analysis is

a preferable way to manage missing data in a cross-sectional study, variables with a missing value greater than 5% in explanatory variables were removed from further analysis. To guarantee quality, the data extractions were carried out by researchers with prior experience working with DHS data.

Model building for multi-level analysis

A multilevel logistic regression model was fitted because the DHS data is hierarchical and children were nested within a cluster, which goes against the independence and equal variance assumptions of ordinary logistic regression models. For the multi-level analysis, four models were fitted. The null model, which solely included the outcome variable, is employed to examine the variation in MDD throughout the cluster. Individual-level and community-level variables are included in the first (Model I) and second (Model II) multilevel models, respectively. The third model (Model III) fitted MDD simultaneously with factors at the individual and community levels. The log-likelihood and deviance test was used to compare the models with the standard logistics regression model, and the best-fitting model was determined by taking the highest log-likelihood and lowest deviance values of each model into account. Multicollinearity was checked using the variance inflation factor (VIF), and a variable is considered to be multi-collinear if its VIF score is 10 or more (31). However, in this investigation, the mean VIF value of the final model was 1.60, and all variables had VIF values less than three. Finally, variables with a p-value of < 0.05 and 95% confidence intervals were declared statistically significant. Proportional Change in Variance (PCV), Intra-class Correlation Coefficient (ICC), and Median Odds Ratio (MOR) were used to evaluate the random effect utilized to measure the variation (32).

Ethical statement

Permission was granted to download and use the data from http://www.dhs.program.com before conducting the study. Ethical clearance was obtained from the Institution Review Board of the DHS Program, ICF International. The procedures for DHS public-use data sets were approved by the Institution Review Board. Identifiers for respondents, households, or sample communities were not allowed in any way, and the names of individuals or household addresses were not included in the data files. The number for each EA in the data file does not have labels to show their names or locations. There were no patients or members of the public involved since this study used a publicly available data set.

Results

Individual- and community-level characteristics of study subjects

The mean age of mothers was 28.12 ± 0.13 years, and 42.27% of them fall in the age range of 25–34 years. More than half (54.79%) of mothers had no formal education, and only 5.51% of them were unmarried. More than three-fourths (79.33%) of mothers were from male-headed households, and 61.07% of them had three or fewer children ever born. More than half (57.83%) of mothers had poor household wealth status, and 82.57% of them had media exposure. More than two-thirds (67.91%) of mothers had four or more ANC visits during their pregnancy, and 81.97% of them currently breastfed their children. The majority (90.52%) of mothers gave birth at a health facility, and 57.77% of them had no postnatal checkup. The mean age of children was 14.18 ± 0.09 months, and 38.09% of them fall in the age range of 12–17 months. More than half (51.59%) of children were male, and 24.54% of them were first-born children. The preceding birth interval for almost all children (99.40%) was ≥ 24 months, and 55.73% of mothers had three or more under-five children in the household. More than one-third (35.43%) of mothers reside in urban areas, and 50.62% of them were from communities with low levels of literacy. Nearly two-thirds (66.58%)

and 55.16% of mothers were from communities with high levels of media exposure and low levels of poverty, respectively [Table 1].

Prevalence of adequate minimum dietary diversity among children aged 6–23 months

In the present study, the proportion of children aged 6-23 months who meet minimum dietary diversity in Senegal was 23.61% (95% CI: 22.12, 25.16) [Figure 2~~1~~]. The highest proportion of children who meet MDD were found in the Saint-Louis region (45.12%) and the lowest in the Sedhiou region (9.84%) [Figure 3~~2~~). The proportion of MDD was 89.68%, 31.40%, 73.27%, 79.92%, and 48.23% among children who had media exposure, rich households, attended 4+ ANC visits, currently breastfeeding, and aged 12-17 months, respectively [Table 2]. From the eight defined groups, breastmilk was consumed by nearly 81% of young children, and only 6.61% of them consumed eggs [Figure 4~~3~~].

Should have the results of bivariate analysis between all independent variables and dependent variables that show a bivariate relationship, such as the higher the maternal education, the higher the percentage of children who met MDD.

Multi-level analysis of factors associated with MDD

Model comparison and random effect analysis

Table ~~2~~3 illustrates that model III in the multilevel analysis outperforms all other multilevel models and the standard logistics regression model (i.e., the model that included all variables but did not include random effect), as it has the highest log likelihood (-1414.7885) and the lowest deviance (2,830) value. According to the ICC value in the null model, cluster differences were responsible for nearly 14.45% of the variation in MDD among children. The MOR between the higher and lower areas among clusters was 2.03, as shown by the MOR in the null model. Furthermore, both individual and community-level variables accounted for around 21% of the heterogeneity in MDD among children [Table 3~~2~~].

Any explanation about the multilevel analysis results here regarding the outcome dependence within the same communities?

Fixed effect analysis

In the final fitted model of multilevel logistics regression analysis (model III), the following factors like media exposure, household wealth index, ANC visits, current breastfeeding status, age of the child, number of under-5 children in the household, and community-level media exposure were significantly associated with MDD. Accordingly, the odds of meeting MDD were 1.51 times higher among mothers who were exposed to media compared with those who weren't [AOR = 1.51; 95% CI (1.08, 2.13)]. Mothers with middle and rich wealth status were 1.58 and 1.84 times more likely to meet MDD than those with poor wealth status, respectively [AOR = 1.58; 95% CI (1.12, 2.24)] and [AOR = 1.84; 95% CI (1.24, 2.74)]. Mothers who attended four or more ANC visits were 1.50 times more likely to meet MDD than those who didn't attend ANC visits [AOR = 1.50; 95% CI (1.05, 2.13)]. Breastfeeding children were 1.82 times more likely to achieve MDD compared with their counterparts [AOR = 1.82; 95% CI (1.37, 2.43)]. Children aged 12–17 months and 18–23 months were 5.21 and 7.47 times more likely to achieve MDD than those aged 6–11 months, respectively [AOR = 5.21; 95% CI (3.94, 6.87)] and [AOR = 7.47; 95% CI (5.43, 10.3)]. Mothers who had no under-five children in the household were 3.97 times higher to meet MDD than those who had three or more under-five children [AOR = 3.97; 95% CI (1.38, 11.4)]. Mothers from communities with high levels of media exposure were 1.41 times more likely to meet MDD compared with their counterparts [AOR = 1.41; 95% CI (1.03, 1.93)] [Table 4~~3~~].

Discussion

The long-term burden of stunting among newborns and young children can be significantly decreased by interventions aimed at improving dietary diversity, which determines the nutritional status of children and is a key predictor of stunting (33, 34). In the present study, the proportion of adequate MDD among children aged 6–23 months in Senegal was 23.61% (95% CI: 22.12, 25.16). This finding is consistent with studies conducted in India (22.46%) (35), 32 sub-Saharan African

countries (25.1%) (13), and three sub-Saharan African countries (23.2%) (21). On the other hand, the current finding is higher than studies conducted in Ethiopia (13.4% & 14.9%) (22, 36) and Pakistan (20%) (24). However, this finding is lower than studies conducted in the Gedeo zone, Ethiopia (29.9%) (25) and Bangladesh (38%) (23). The possible justification for this similarity and discrepancy can be explained by variations in geographical location, study period, seasonal variability, cultural variation, sample size, and other socioeconomic factors.

The study also identified individual and community-level factors significantly associated with MDD. Accordingly, the odds of meeting MDD were higher among mothers who were exposed to media compared with those who weren't. Likewise, mothers from communities with high levels of media exposure were more likely to meet MDD. This finding is in agreement with studies conducted in Ethiopia (22, 36) and three sub-Saharan African countries (21). The media that women are exposed to, such as reading newspapers, watching TV, and listening to the radio, can encourage them to use maternal healthcare facilities (37, 38). Mass media's informative, instrumental, social control, and community roles in health communication might influence attitudes toward changes in healthy behavior (39). Children from wealthier households were more likely to achieve MDD. This finding is consistent with studies conducted in Ethiopia (25, 36), three sub-Saharan African countries (21), and Bangladesh (23). This may be explained by the high cost of a variety of foods and the idea that eating them is a luxury. Rather than providing their children with a variety of foods, mothers from low-income backgrounds may also sell their animals and goods in order to get enough money to pay for their children's education, food, and other needs.

Mothers who attended four or more ANC visits were more likely to meet MDD than those who didn't. This finding is in line with studies conducted in Indonesia (26) and South Asia (27). This may be the result of mothers' increased awareness of suitable child feeding practices brought about

by the information and counseling they receive from health care providers while using ANC services. This suggests that in order to enhance the eating behaviors of infants and young children, the usage of maternity services should be encouraged. Breastfeeding children were more likely to achieve MDD compared with their counterparts. This finding is in agreement with a study conducted in Brazil (40). A plausible explanation for the correlation between extended breastfeeding and improved feeding practices in later childhood is that families who adhere to the advice to continue breastfeeding for a minimum of two years, without providing non-human milk are also likely to prioritize other dietary recommendations for a healthy lifestyle (41).

Likewise, older children were more likely to achieve MDD than younger ones. This finding is consistent with studies conducted in the Gedeo zone, Ethiopia (25), three sub-Saharan African countries (21), and Pakistan (24). The fact that older children are more accustomed to food than younger children and are more willing to consume a variety of foods with varying tastes and textures could be one explanation for this link (26). In addition, this outcome may be the result of mothers generally delaying the introduction of cereals and legumes to their very young children (6–11 months) until the child turns one year old (42). Mothers who had no under five children in the household were more likely to meet MDD than those who had three or more under-five children. This finding is in line with a study conducted in Ethiopia (43). One explanation could be because mothers with large numbers of under five children are less likely to buy a variety of food categories and are therefore unable to provide for their children's nutritional needs.

Strengths and limitations of the study

The main strength of the study is utilizing weighted nationally representative data with a large sample size, which makes it representative at the national level. The present study also has sufficient statistical power to generalize the estimates of MDD among children aged 6 to 23 months

during the research period. On the other hand, recall and social desirability bias are likely to arise due to the cross-sectional nature of the data collection method (self-reported). The secondary nature of data was also another limitation of this study, because key variables that affect MDD like local agriculture, distance to major cities, seasonality, and local climate were not incorporated in the current analysis.

Conclusion

Nearly one in four children aged 6 to 23 months achieved minimum dietary diversity in Senegal. Children from mothers with media exposure, wealthier households, attended 4+ ANC visits, are currently breastfeeding, older children, and no under-5 children in the household were associated with higher odds of meeting MDD. Therefore, improving media access, women empowerment, enhancing ANC service utilization, encouraging continued breastfeeding, and giving prior attention to younger children aged 6 to 11 months and mothers who had under-five children in the household are strongly recommended to improve feeding practices of infant and young children.

List of abbreviations: ANC: Antenatal Care; AOR: Adjusted Odds Ratio; CI: Confidence Interval; DHS: Demographic and Health Survey; ICC: Intra-class Correlation Coefficient; MDD: Minimum Dietary Diversity; MOR: Median Odds Ratio; PCV: Proportional Change in Variance; VIF: Variance Inflation Factor; WHO: World Health Organization

Consent for publication: Not applicable.

Availability of data and materials: The data from the DHS is publicly available online at https://dhsprogram.com/data/available-datasets.cfm.

Competing interest: The author declared that there was no competing interest.

Funding: Not applicable

Author contributions: Conceptualization, data curation, formal analysis, investigation, methodology, software, visualization, writing the original draft, reviewing, and editing were done by E.G.M.

Acknowledgments: I am grateful to the DHS program for letting me use the relevant data in this study.

References

1. UNICEF. Early childhood nutrition; Preventing malnutrition in infants and young children. 2024.
2. UNICEF. Improving young children’s diets during the complementary feeding period. UNICEF programming guidance. 2020;76:118-22.2023.
3. Dewey K. Guiding principles for complementary feeding of the breastfed child. 2003.
4. Organization WH. Guiding principles for feeding non-breastfed children 6-24 months of age: World Health Organization; 2005.
5. Onyango AW, Borghi E, de Onis M, del Carmen Casanovas M, Garza C. Complementary feeding and attained linear growth among 6–23-month-old children. Public health nutrition. 2014;17(9):1975-83.
6. Prado EL, Dewey KG. Nutrition and brain development in early life. Nutrition reviews. 2014;72(4):267-84.
7. Aguayo VM, Nair R, Badgaiyan N, Krishna V. Determinants of stunting and poor linear growth in children under 2 years of age in India: An in-depth analysis of Maharashtra's comprehensive nutrition survey. Maternal & child nutrition. 2016;12:121-40.
8. Leal KK, Schneider BC, França GVA, Gigante DP, Santos Id, Assunção MCF. Diet quality of preschool children aged 2 to 5 years living in the urban area of Pelotas, Brazil. Revista Paulista de Pediatria. 2015;33:310-7.
9. Zhou Z, Liew J, Yeh Y-C, Perez M. Appetitive traits and weight in children: evidence for parents’ controlling feeding practices as mediating mechanisms. The Journal of genetic psychology. 2020;181(1):1-13.
10. Lutter CK, Grummer-Strawn L, Rogers L. Complementary feeding of infants and young children 6 to 23 months of age. Nutrition Reviews. 2021;79(8):825-46.
11. Bhandari N. Infant and young child feeding. 2016.
12. Organization WH. Indicators for assessing infant and young child feeding practices: definitions and measurement methods. 2021.
13. Aboagye RG, Seidu A-A, Ahinkorah BO, Arthur-Holmes F, Cadri A, Dadzie LK, et al. Dietary diversity and undernutrition in children aged 6–23 months in Sub-Saharan Africa. Nutrients. 2021;13(10):3431.
14. Kennedy GL, Pedro MR, Seghieri C, Nantel G, Brouwer I. Dietary Diversity Score Is a Useful Indicator of Micronutrient Intake in Non-Breast-Feeding Filipino Children2. The Journal of nutrition. 2007;137(2):472-7.
15. McDonald CM, Olofin I, Flaxman S, Fawzi WW, Spiegelman D, Caulfield LE, et al. The effect of multiple anthropometric deficits on child mortality: meta-analysis of individual data in 10 prospective studies from developing countries. The American journal of clinical nutrition. 2013;97(4):896-901.

Field Code Changed

Formatted: Font: (Default) Times New Roman

16. Khamis AG, Mwanri AW, Ntwenya JE, Kreppel K. The influence of dietary diversity on the nutritional status of children between 6 and 23 months of age in Tanzania. BMC pediatrics. 2019;19:1-9.
17. Saha J, Chouhan P, Malik NI, Ghosh T, Das P, Shahid M, et al. Effects of Dietary Diversity on Growth Outcomes of Children Aged 6 to 23 Months in India: Evidence from National Family and Health Survey. Nutrients. 2022;15(1):159.
18. Ballini L. Food Insecurity and Lack of Dietary Diversity: Its Association with Anemia In Senegalese Women and Children.
19. Janmohamed A, Baker MM, Doledec D, Ndiaye F, Konan ACL, Leonce A, et al. Dietary diversity and associated factors among infants and young children in three West African countries. Frontiers in Public Health. 2024;12:1386664.
20. POUYE R, Ndicak F. Nutritional status and associated factors in children under five in Senegal: an empirical analysis. Moroccan Journal of Public Heath. 2023;5(1).
21. Ba DM, Ssentongo P, Gao X, Chinchilli VM, Richie Jr JP, Maiga M, et al. Prevalence and determinants of meeting minimum dietary diversity among children aged 6–23 months in three sub-Saharan African Countries: The Demographic and Health Surveys, 2019–2020. Frontiers in Public Health. 2022;10:846049.
22. Eshete T, Kumera G, Bazezew Y, Mihretie A, Marie T. Determinants of inadequate minimum dietary diversity among children aged 6–23 months in Ethiopia: secondary data analysis from Ethiopian Demographic and Health Survey 2016. Agriculture & Food Security. 2018;7(1):1-8.
23. Khanam M, Sarker AR. Dietary Diversity among Children Aged 6-23 Months in Bangladesh. The Bangladesh Development Studies. 2021;44(3/4):81-102.
24. Kumar R, Mahmood T, Naeem N, Khan SA, Hanif M, Pongpanich S. Minimum dietary diversity and associated determinants among children aged 6–23 months in Pakistan. Scientific Reports. 2024;14(1):2639.
25. Molla W, Adem DA, Tilahun R, Shumye S, Kabthymer RH, Kebede D, et al. Dietary diversity and associated factors among children (6–23 months) in Gedeo zone, Ethiopia: cross-sectional study. Italian journal of pediatrics. 2021;47(1):233.
26. Paramashanti BA, Huda TM, Alam A, Dibley MJ. Trends and determinants of minimum dietary diversity among children aged 6–23 months: a pooled analysis of Indonesia demographic and health surveys from 2007 to 2017. Public health nutrition. 2022;25(7):1956-67.
27. Rahman MA, Kundu S, Rashid HO, Tohan MM, Islam MA. Socio-economic inequalities in and factors associated with minimum dietary diversity among children aged 6–23 months in South Asia: a decomposition analysis. BMJ open. 2023;13(12):e072775.
28. National Agency for Statistics and Demography (NASD) [Senegal] aI. Demographic Survey and Continuing Health (2023 Continuous Survey) Maryland, USA: NASD and ICF. 2023.
29. Macro O. Demographic and health surveys. Calverton, MD: ICF Macro. 2004.
30. Croft TN, Allen, Courtney K., Zachary, Blake W., et al. Guide to DHS Statistics. Rockville, Maryland, USA: ICF. 2023.
31. Daoud JI, editor Multicollinearity and regression analysis. Journal of Physics: Conference Series; 2017: IOP Publishing.
32. Sommet N, Morselli D. Keep calm and learn multilevel logistic modeling: A simplified three-step procedure using Stata, R, Mplus, and SPSS. International Review of Social Psychology. 2017;30:203-18.
33. Ahmad I, Khalique N, Khalil S, Maroof M. Dietary diversity and stunting among infants and young children: a cross-sectional study in Aligarh. Indian Journal of Community Medicine. 2018;43(1):34-6.
34. Borkotoky K, Unisa S, Gupta AK. State-level dietary diversity as a contextual determinant of nutritional status of children in India: a multilevel approach. Journal of biosocial science. 2018;50(1):26-52.
35. Padigapati VNS, Singh A, KV P. Are children attaining the minimum dietary diversity criteria in India? Evidence from National Family Health Survey-5. 2024.

36. Mulaw GF, Mohammed OA, Mare KU. Level of dietary diversity score and its predictors among children aged 6–23 months: a linear mixed model analysis of the 2019 Ethiopian Mini Demographic Health Survey. BMJ Public Health. 2024;2(1).

37. Aboagye RG, Seidu A-A, Ahinkorah BO, Cadri A, Frimpong JB, Hagan JE, et al. Association between frequency of mass media exposure and maternal health care service utilization among women in sub-Saharan Africa: implications for tailored health communication and education. Plos one. 2022;17(9):e0275202.

38. Fatema K, Lariscy JT. Mass media exposure and maternal healthcare utilization in South Asia. SSM-Population Health. 2020;11:100614.

39. Sarrassat S, Meda N, Ouedraogo M, Some H, Bambara R, Head R, et al. Behavior change after 20 months of a radio campaign addressing key lifesaving family behaviors for child survival: midline results from a cluster randomized trial in rural Burkina Faso. Global Health: Science and Practice. 2015;3(4):557-76.

40. Bortolini GA, Giugliani ERJ, Gubert MB, Santos LMP. Breastfeeding is associated with children's dietary diversity in Brazil. Ciência & Saúde Coletiva. 2019;24:4345-54.

41. Vitolo MR, Bortolini GA, Campagnolo PDB, Hoffman DJ. Maternal dietary counseling reduces consumption of energy-dense foods among infants: a randomized controlled trial. Journal of nutrition education and behavior. 2012;44(2):140-7.

42. Baek Y, Chitekwe S. Sociodemographic factors associated with inadequate food group consumption and dietary diversity among infants and young children in Nepal. PloS one. 2019;14(3):e0213610.

43. Woldegebriel AG, Desta AA, Gebreegziabiher G, Berhe AA, Ajemu KF, Woldearegay TW. Dietary Diversity and Associated Factors among Children Aged 6-59 Months in Ethiopia: Analysis of Ethiopian Demographic and Health Survey 2016 (EDHS 2016). International journal of pediatrics. 2020;2020(1):3040845.

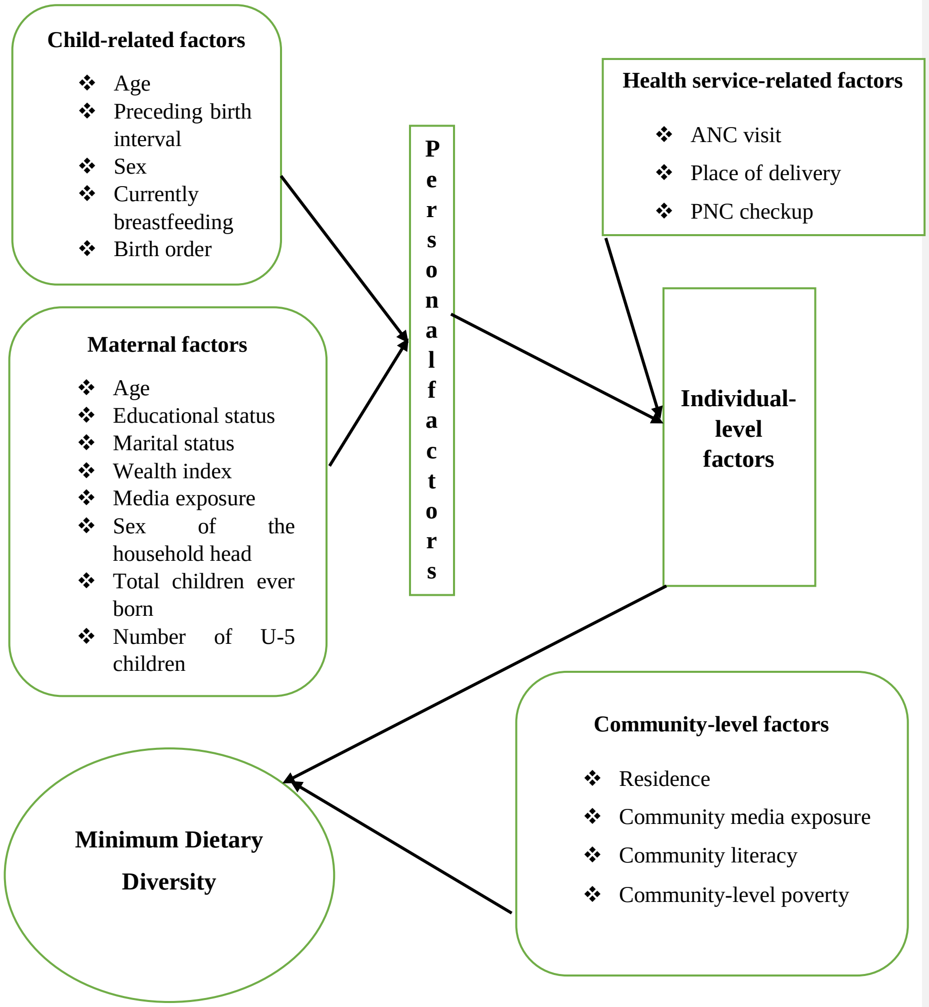

Figure 1: Conceptual framework for factors associated with minimum dietary diversity among children aged 6 to 23 months in Senegal

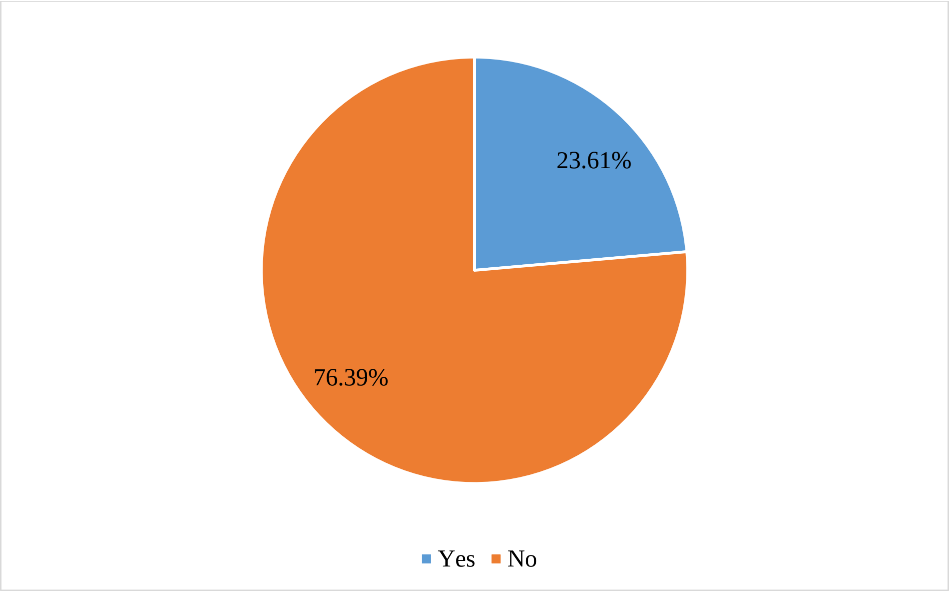

Figure 21: Proportion of children aged 6-23 months who meet minimum dietary diversity in Senegal; DHS 2023 (n = 2,995)

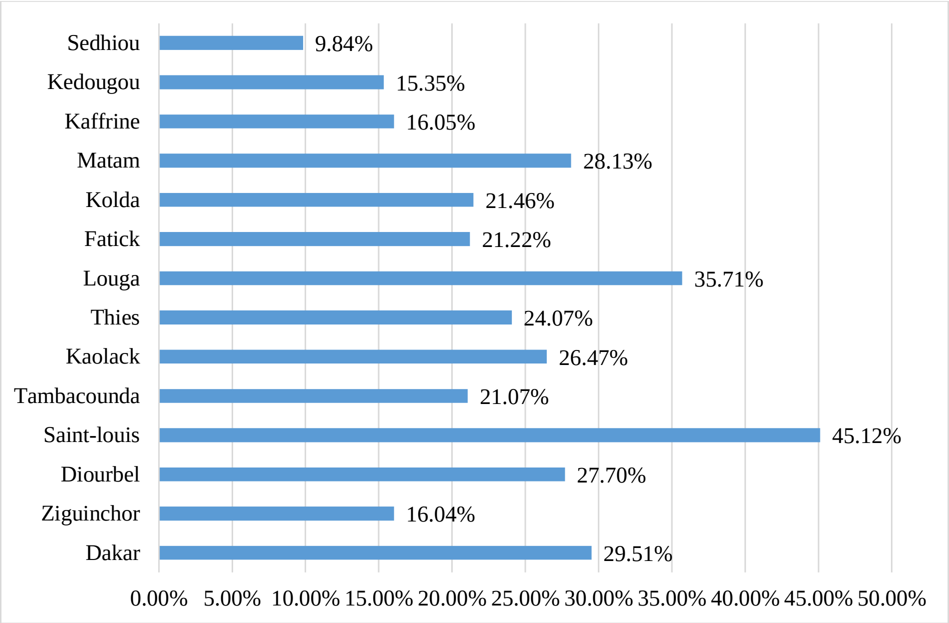

Figure 32: Regional proportion of children aged 6-23 months who meet minimum dietary diversity in Senegal; DHS 2023 (n = 2,995)

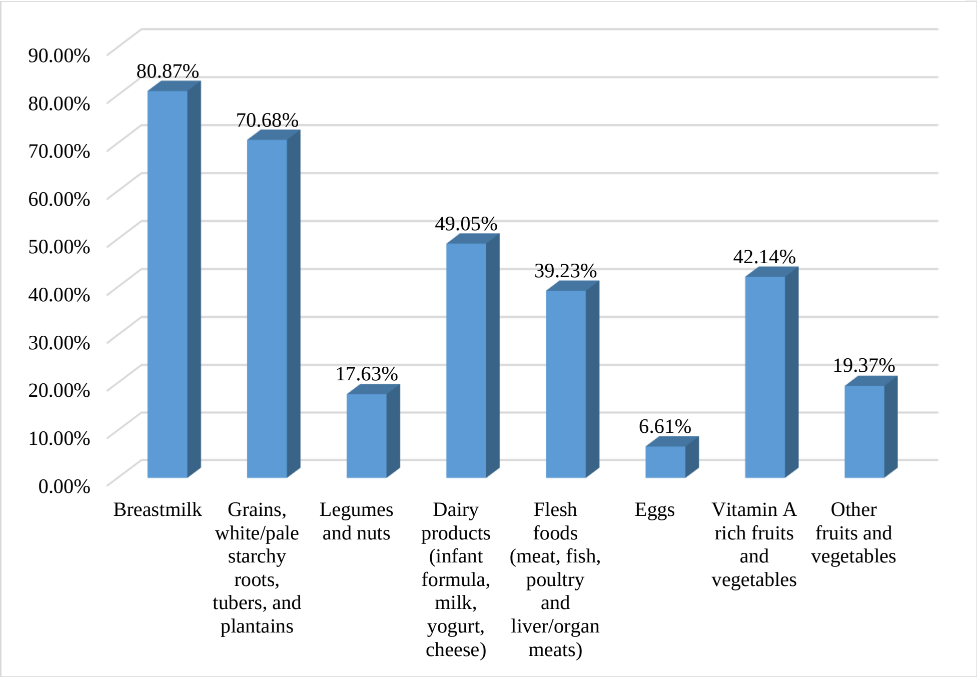

Figure 43: Proportion of children aged 6-23 months who consumed the eight defined food groups in Senegal; DHS 2023 (n = 2,995)

Table 1: Individual-and community-level characteristics of study subjects in Senegal; DHS 2023

Variables	Category	Frequency (n)	Percentage (%)
Maternal age	15–24 years	1,083	36.16
	25–34 years	1,266	42.27
	35–49 years	646	21.57
Maternal education	No education	1,641	54.79
	Primary	564	18.83
	Secondary & higher	790	26.38
Marital status	Unmarried	165	5.51
	Married	2,830	94.49
Sex of the household head	Male	2,376	79.33
	Female	619	20.67
Total children ever born	1-3	1,829	61.07
	4-6	849	28.35
	≥7	317	10.58
Media exposure	No	522	17.43
	Yes	2,473	82.57
Wealth index	Poor	1,732	57.83
	Middle	562	18.76
	Rich	701	23.41
ANC visits	None	295	9.85
	1-3 visits	666	22.24
	4+ visits	2,034	67.91
Place of delivery	Home	284	9.48
	Health facility	2,711	90.52
Currently breastfeeding	No	540	18.03
	Yes	2,455	81.97
PNC checkup	No	1,677	57.77
	Yes	1,226	42.23
Sex of the child	Male	1,545	51.59
	Female	1,450	48.41

Age of the child	6–11 months	982	32.79
	12–17 months	1,141	38.09
	18–23 months	872	29.12
Birth order	First-born	735	24.54
	2-4	1,466	48.95
	5+	794	26.51
Preceding birth interval	< 24 months	18	0.60
	≥ 24 months	2,977	99.40
Under-5 children in the household	None	20	0.67
	1–2	1,306	43.60
	3+	1,669	55.73
Place of residence	Rural	1,934	64.57
	Urban	1,061	35.43
Community literacy	Low	1,516	50.62
	High	1,479	49.38
Community poverty level	low	1,652	55.16
	High	1,343	44.84
Community media exposure	Low	1,001	33.42
	High	1,994	66.58

Table 2: Model comparison and random effect analysis for minimum dietary diversity and its associated factors among women in Senegal; DHS 2023 (n = 2,995)

Parameter	Null model	Model I	Model II	Model III
Variance	0.555578	0.4460063	0.4114763	0.437621
ICC	14.45%	11.94%	11.12%	11.74%
MOR	2.03	1.88	1.84	1.87
PCV	Reference	19.72%	25.94%	21.23%
**Model fitness**				
LLR	-1608.4565	-1417.4869	-1588.1932	-1414.7885
Deviance	3,216.913	2,834.9738	3,176.3864	2,829.577

ICC: Intra cluster correlation; LLR: Log-likelihood ratio; MOR: Median odds ratio; PCV: Proportional change in variance

Table 3: Multivariable multilevel logistic regression analysis of factors associated with minimum dietary diversity among children in Senegal; DHS 2023 (n = 2,995)

Variables	Category	Model I AOR (95% CI)	Model II AOR (95% CI)	Model III AOR (95% CI)
Maternal age	15–24 years	1.00		1.00
	25–34 years	1.05 (0.81, 1.37)		1.05 (0.81, 1.37)
	35–49 years	0.89 (0.62, 1.30)		0.89 (0.62, 1.30)
Maternal education	No education	1.00		1.00
	Primary	0.92 (0.70, 1.20)		0.91 (0.69, 1.19)
	Secondary & higher	0.85 (0.65, 1.11)		0.83 (0.63, 1.11)
Marital status	Unmarried	1.00		1.00
	Married	1.18 (0.74, 1.87)		1.19 (0.75, 1.89)
Sex of the household head	Male	1.00		1.00
	Female	1.09 (0.86, 1.39)		1.08 (0.85, 1.38)
Total children ever born	1-3	0.76 (0.44, 1.31)		0.76 (0.44, 1.31)
	4-6	0.75 (0.50, 1.13)		0.75 (0.50, 1.13)
	≥7	1.00		1.00
Media exposure	No	1.00		1.00
	Yes	1.74(1.26, 2.39)*		1.51(1.08, 2.13)*
Wealth index	Poor	1.00		1.00
	Middle	1.67(1.27, 2.21)*		1.58(1.12, 2.24)*
	Rich	2.01(1.51, 2.69)*		1.84(1.24, 2.74)*
ANC visits	None	1.00		1.00
	1-3 visits	1.18 (0.79, 1.76)		1.19 (0.80, 1.77)
	4+ visits	1.50(1.05, 2.13)*		1.50(1.05, 2.13)*
Place of delivery	Home	1.00		1.00
	Health facility	1.16 (0.79, 1.71)		1.13 (0.77, 1.66)
Currently breastfeeding	No	1.00		1.00
	Yes	1.82(1.37, 2.43)*		1.82(1.37, 2.43)*
PNC checkup	No	1.00		1.00
	Yes	1.07 (0.87, 1.31)		1.07 (0.87, 1.31)

Sex of the child	Male	1.00		1.00
	Female	1.05 (0.87, 1.28)		1.05 (0.87, 1.28)
Age of the child	6–11 months	1.00		1.00
	12–17 months	5.21(3.95, 6.88)*		5.21(3.94, 6.87)*
	18–23 months	7.46(5.43, 10.3)*		7.47(5.43, 10.3)*
Birth order	First-born	0.86 (0.54, 1.38)		0.86 (0.54, 1.38)
	2-4	0.89 (0.62, 1.29)		0.89 (0.62, 1.29)
	5+	1.00		1.00
Preceding birth interval	< 24 months	1.00		1.00
	≥ 24 months	0.61 (0.19, 2.03)		0.61 (0.18, 2.03)
Under-5 children in the household	None	4.13(1.44, 11.8)*		3.97(1.38, 11.4)*
	1–2	1.17 (0.96, 1.44)		1.17 (0.95, 1.44)
	3+	1.00		1.00
Place of residence	Rural		1.00	1.00
	Urban		1.24(0.91, 1.68)	1.10 (0.79, 1.53)
Community literacy	Low		1.00	1.00
	High		1.00(0.78, 1.28)	1.06 (0.80, 1.40)
Community poverty level	low		0.77(0.56, 1.06)	1.09 (0.72, 1.63)
	High		1.00	1.00
Community media exposure	Low		1.00	1.00
	High		1.67(1.26,2.20)*	1.41(1.03, 1.93)*

*Statistically significant at P-value<0.05; ANC: Antenatal care; PNC: Postnatal care

Response to reviewers

Reviewer	Comments	Author's response
Reviewer 1	What is the outcome variable?	Thank you very much and I have corrected it.
	Meeting the minimum dietary diversity?	Corrected.
	Higher odds of meeting minimum dietary diversity?	Corrected.
	Please also support with the statement from the Government of Senegal that it is imperative.	Considered.
	Before moving to the objective of the study, provide with the theoretical framework and more results of empirical studies on the determinants of MDD as well the novelty of the study.	I have included determinants of MDD from previous studies and a conceptual framework.
	Why proportion? Isn't that it is the MDD status of a child which is binary, adequate or inadequate?	Corrected.
	Provide also the bivariate analyses of the proportion of children who meet MDD by other independent variables in the models in order to investigate the patterns and differentials of MDD by background characteristics.	Corrected.
	Please provide with the statistics that measure and confirm the significance of community level variable (in form of scale parameters).	Considered.
Reviewer 2	It is unclear what the contribution of the paper is to the literature on dietary diversity for children in Senegal.<br>a) Factors that determine minimum dietary diversity for children in Senegal, as well as Sub-	Repeated studies are essential to know whether there is improvement in child health or not and to understand the time variation that give insight for policymakers and program

	Saharan Africa, are well documented (see for e.g., Thorne-Lyman et al. 2020; Aboagye et al. 2021). Is the contribution of this paper that it utilizes the most recent DHS data? If so, the manuscript would benefit from discussions on how dietary diversity in Senegal has changed, and what new information can be identified using this most recent survey.	managers. Using recent data are also significant to understand the status of child nutrition and intervene accordingly.
	b) Or, is the motivation that the paper identifies both individual and community level media exposure as a key determinant of children having adequate minimum dietary diversity? Linking media exposure to dietary diversity is relatively new for Senegal but has been explored for Ethiopia (Jalata and Asefa 2022) and other Sub-Saharan countries (Belay et al. 2022). Discussions on why media exposure is a key factor for dietary diversity need to be made clear in both the Introduction and Results sections.	This an indicator of the importance of information dissemination through media to improve the nutritional status of children, including meeting MDD. Countries should invest on accessing media including radio, newspaper, and television.
	The key attribute of this paper is identifying the variables significantly linked to children with adequate minimum dietary diversity. While the paper does use a comprehensive set of individual and community level characteristics, these are not exhaustive. For instance, mother's occupation, father's characteristics, local agriculture, distance to major cities, and local climate are some key variables that could also impact minimum dietary diversity. Models don't have to include all possible variables, but a better	Accessing all variables from DHS is not possible and this is mentioned under the limitations of the study section.

	explanation of the choice of independent variables needs to be provided. At the moment, the paper lacks justification for why certain variables were chosen, and more importantly, why others were not.	
	On a similar note, defining poverty levels for community by taking 50% of women who belonged to the lowest and worst wealth quintiles is also not justified. This seems like an arbitrary number that might not really reflect poverty levels in Senegal. Justification for this would also help readers understand subsequent results.	Corrected.
	Media exposure is defined as members having access to newspapers, watching television, or listening to the radio (line 147). These variables seem to be strongly correlated with household wealth and access to basic amenities. The fact that media exposure is strongly associated with adequate minimum dietary diversity is not surprising, as its likely that households with media exposure are simply wealthier households. At the moment, both the wealth index and media exposure are significant, but I am concerned that these two variables merely reflect wealthier households. A stronger justification is needed to explain why these two variables are distinct.<br>What is specific about 'media exposure' that explains why children achieve minimum dietary diversity that is not related to household wealth index?	Media exposure is not directly related with household wealth status. Because poorer households can have media exposure from their friends or neighbors regardless of their wealth status.

	Regarding the empirical methods, the paper assesses model strength using log-likelihood (line 179). This value is further used to justify the selection of models (line 225). However, to the best of my knowledge, log-likelihood values improve with more variables. Therefore, the fact that Model III (Table 2) has the lowest log-likelihood is not surprising, as Model III includes the highest number of independent variables. As in Comment #2, better justification for the choice of model is needed. Why is Model III considered rather than any alternative model with more additional variables?	Model III has the highest log likelihood not lowest.<br>The model with the highest log likelihood (-1414.7885) and the lowest deviance (2,830) value was selected as best fitted model (Model III).
	Recent studies have highlighted that in Senegal, seasonality plays a key role in determining a child's dietary diversity (see for e.g., Shakya, Bevis and Thorne-Lyman 2024; Thorne-Lyman et al. 2021). During the harvest season, households have higher incomes and food availability, leading to better child diets. Similarly, Ramadan is a key festival in Senegal, which directly affects household diets. Information on when the DHS surveys were conducted (e.g., how many households were surveyed in Jan, Feb… Dec), would help readers understand how seasonality might bias minimum dietary diversity estimates. For example, if most households were surveyed during the harvest season – when food and income levels are high – then dietary diversity among children in Senegal	As this data is not accessible from the DHS, it is mentioned as limitation of the study.

	is likely overestimated. Discussing this in the Limitation section would be helpful.	
	Line 17: I think the software is "STATA". The manuscript is missing an A.	Corrected.
	Line 42: "caregiver should engage with their children to acknowledge their hunger cues", is a bit unclear. I am not sure what this means.	Corrected.
	Line 49: This sentence talks about the links between stunting and diet. There are many other key papers on this topic and should cite a few more seminal papers on this topic.	Considered.
	Line 73: Similarly, this sentence talks about the importance of animal-sourced food. A lot of work on this has already been done and would benefit from citing these too.	Considered.
	Line 77: MDD was 30.8%. Which year was this data for? The paper's MDD is around 23%. There seems to be a big difference between these two MDD.	The data collection was conducted in 2022 and published in 2024. The study was conducted in three west African countries.
	Line 93: What are EDS surveys? I could not find explanation for this.	Corrected.
	Line 106: To the best of my knowledge, KR stands for Kids Recode.	Corrected.
	Line 254: MDD scores for 32 Sub-Saharan country is 25.1%, but for 3 Sub-Saharan country it is 23.2%. Not sure what this means.	These are results from previous studies and no need of defining this.
	Line 272-274: This claim needs supporting evidence (papers or reports).	Considered.
Thank you very much for your time and effort.		
