# Supplementary material for: Minimum dietary diversity and its associated factors among children aged 6–23 months in Senegal: Evidence from the demographic and health survey, 2023
Source: PLoS One. 2025 Sep 11;20(9):e0332215. doi: 10.1371/journal.pone.0332215 (PMC12425297; doi:10.1371/journal.pone.0332215)
Supplement: Attachment — Submitted filename: Response to Reviewers.docx [file pone.0332215.s003.docx]

**Response to reviewers**

| **Reviewer** | **Comments** | **Author’s response** |
| --- | --- | --- |
| Reviewer 1 | What is the outcome variable? | Thank you very much and I have corrected it. |
|  | Meeting the minimum dietary diversity? | Corrected. |
|  | Higher odds of meeting minimum dietary diversity? | Corrected. |
|  | Please also support with the statement from the Government of Senegal that it is imperative. | Considered. |
|  | Before moving to the objective of the study, provide with the theoretical framework and more results of empirical studies on the determinants of MDD as well the novelty of the study. | I have included determinants of MDD from previous studies and a conceptual framework. |
|  | Why proportion? Isn’t that it is the MDD status of a child which is binary, adequate or inadequate? | Corrected. |
|  | Provide also the bivariate analyses of the proportion of children who meet MDD by other independent variables in the models in order to investigate the patterns and differentials of MDD by background characteristics. | Corrected. |
|  | Please provide with the statistics that measure and confirm the significance of community level variable (in form of scale parameters). | Considered. |
| Reviewer 2 | It is unclear what the contribution of the paper is to the literature on dietary diversity for children in Senegal.  a) Factors that determine minimum dietary diversity for children in Senegal, as well as Sub-  Saharan Africa, are well documented (see for e.g., Thorne-Lyman et al. 2020; Aboagye et  al. 2021). Is the contribution of this paper that it utilizes the most recent DHS data? If so, the manuscript would benefit from discussions on how dietary diversity in Senegal has changed, and what new information can be identified using this most recent survey. | Repeated studies are essential to know whether there is improvement in child health or not and to understand the time variation that give insight for policymakers and program managers. Using recent data are also significant to understand the status of child nutrition and intervene accordingly. |
|  | b) Or, is the motivation that the paper identifies both individual and community level media exposure as a key determinant of children having adequate minimum dietary diversity? Linking media exposure to dietary diversity is relatively new for Senegal but has been explored for Ethiopia (Jalata and Asefa 2022) and other Sub-Saharan countries (Belay et  al. 2022). Discussions on why media exposure is a key factor for dietary diversity need to be made clear in both the Introduction and Results sections. | This an indicator of the importance of information dissemination through media to improve the nutritional status of children, including meeting MDD. Countries should invest on accessing media including radio, newspaper, and television. |
|  | The key attribute of this paper is identifying the variables significantly linked to children with adequate minimum dietary diversity. While the paper does use a comprehensive set of individual and community level characteristics, these are not exhaustive. For instance, mother’s occupation, father’s characteristics, local agriculture, distance to major cities, and local climate are some key variables that could also impact minimum dietary diversity. Models don’t have to include all possible variables, but a better explanation of the choice of independent variables needs to be provided. At the moment, the paper lacks justification for why certain variables were chosen, and more importantly, why others were not. | Accessing all variables from DHS is not possible and this is mentioned under the limitations of the study section. |
|  | On a similar note, defining poverty levels for community by taking 50% of women who belonged to the lowest and worst wealth quintiles is also not justified. This seems like an arbitrary number that might not really reflect poverty levels in Senegal. Justification for this would also help readers understand subsequent results. | Corrected. |
|  | Media exposure is defined as members having access to newspapers, watching television, or listening to the radio (line 147). These variables seem to be strongly correlated with household wealth and access to basic amenities. The fact that media exposure is strongly associated with adequate minimum dietary diversity is not surprising, as its likely that households with media exposure are simply wealthier households. At the moment, both the wealth index and media exposure are significant, but I am concerned that these two variables merely reflect wealthier households. A stronger justification is needed to explain why these two variables are distinct.  What is specific about ‘media exposure’ that explains why children achieve minimum dietary diversity that is not related to household wealth index? | Media exposure is not directly related with household wealth status. Because poorer households can have media exposure from their friends or neighbors regardless of their wealth status. |
|  | Regarding the empirical methods, the paper assesses model strength using log-likelihood (line 179). This value is further used to justify the selection of models (line 225). However, to the best of my knowledge, log-likelihood values improve with more variables. Therefore, the fact that Model III (Table 2) has the lowest log-likelihood is not surprising, as Model III includes the highest number of independent variables. As in Comment #2, better justification for the choice of model is needed. Why is Model III considered rather than any alternative model with more additional variables? | Model III has the highest log likelihood not lowest.  The model with the highest log likelihood (-1414.7885) and the lowest deviance (2,830) value was selected as best fitted model (Model III). |
|  | Recent studies have highlighted that in Senegal, seasonality plays a key role in determining a child’s dietary diversity (see for e.g., Shakya, Bevis and Thorne-Lyman 2024; Thorne-Lyman et al. 2021). During the harvest season, households have higher incomes and food availability, leading to better child diets. Similarly, Ramadan is a key festival in Senegal, which directly affects household diets. Information on when the DHS surveys were conducted (e.g., how many households were surveyed in Jan, Feb… Dec), would help readers understand how seasonality might bias minimum dietary diversity estimates. For example, if most households were surveyed during the harvest season – when food and income levels are high – then dietary diversity among children in Senegal is likely overestimated. Discussing this in the Limitation section would be helpful. | As this data is not accessible from the DHS, it is mentioned as limitation of the study. |
|  | Line 17: I think the software is “STATA”. The manuscript is missing an A. | Corrected. |
|  | Line 42: “caregiver should engage with their children to acknowledge their hunger cues”, is a bit unclear. I am not sure what this means. | Corrected. |
|  | Line 49: This sentence talks about the links between stunting and diet. There are many other key papers on this topic and should cite a few more seminal papers on this topic. | Considered. |
|  | Line 73: Similarly, this sentence talks about the importance of animal-sourced food. A lot of work on this has already been done and would benefit from citing these too. | Considered. |
|  | Line 77: MDD was 30.8%. Which year was this data for? The paper’s MDD is around 23%. There seems to be a big difference between these two MDD. | The data collection was conducted in 2022 and published in 2024. The study was conducted in three west African countries. |
|  | Line 93: What are EDS surveys? I could not find explanation for this. | Corrected. |
|  | Line 106: To the best of my knowledge, KR stands for Kids Recode. | Corrected. |
|  | Line 254: MDD scores for 32 Sub-Saharan country is 25.1%, but for 3 Sub-Saharan country it is 23.2%. Not sure what this means. | These are results from previous studies and no need of defining this. |
|  | Line 272-274: This claim needs supporting evidence (papers or reports). | Considered. |
| ***Thank you very much for your time and effort.*** | | |
