# Supplementary material for: Minimum dietary diversity and its associated factors among children aged 6–23 months in Senegal: Evidence from the demographic and health survey, 2023
Source: PLoS One. 2025 Sep 11;20(9):e0332215. doi: 10.1371/journal.pone.0332215 (PMC12425297; doi:10.1371/journal.pone.0332215)
Supplement: Attachment — Submitted filename: Response_to_Reviewers_auresp_2.docx [file pone.0332215.s005.docx]

**Response to reviewers**

| **Reviewer** | **Comments** | **Author’s response** |
| --- | --- | --- |
| Reviewer 1 | Use italic for p in p-value | Thank you very much and I have corrected it. |
|  | Still needs to address the general research problem i.e. that in Senegal the negative impact of inadequate minimum dietary diversity, such as lower education achievement, is a problem, usually addressed in the national development plan. | I have addressed it. |
|  | Still needs to address the novelty of this study. | I have addressed it. |
|  | Is there any statistical problem using maternal education and this community problem in the same model? | Yes, because cluster number is incorporated to generate community level variables. |
|  | Should have the results of bivariate analysis between all independent variables and dependent variables that show a bivariate relationship, such as the higher the maternal education, the higher the percentage of children who met MDD. | Thank you very much and I have included the biviriable analysis. |
|  | Any explanation about the multilevel analysis results here regarding the outcome dependence within the same communities? | Thank you very much and I have included an explanation. |
|  | Why mothers? How about the children? | Corrected. |
| ***Thank you very much for your time and effort.*** | | |
